# Peroxidase proximity selection to identify aptamers targeting a subcellular location

**DOI:** 10.1093/pnasnexus/pgad151

**Published:** 2023-05-04

**Authors:** Brandon Wilbanks, William Beimers, Maria Dugan, Taylor Weiskittel, L J Maher

**Affiliations:** Department of Biochemistry and Molecular Biology, Mayo Clinic, Rochester, MN 55905, USA; Department of Biochemistry and Molecular Biology, Mayo Clinic, Rochester, MN 55905, USA; Present address: Department of Biochemistry, University of Wisconsin-Madison, Madison, WI 53706, USA; Department of Biochemistry and Molecular Biology, Mayo Clinic, Rochester, MN 55905, USA; Present address: Department of Chemistry, Iowa State University, Ames, IA 50011, USA; Department of Molecular Pharmacology and Experimental Therapeutics, Mayo Clinic, Rochester, MN 55901, USA; Department of Biochemistry and Molecular Biology, Mayo Clinic, Rochester, MN 55905, USA

**Keywords:** aptamer, oligonucleotide, proximity biotinylation, subcellular targeting

## Abstract

The efficient and specific delivery of functional cargos such as small-molecule drugs, proteins, or nucleic acids across lipid membranes and into subcellular compartments is a significant unmet need in nanomedicine and molecular biology. Systematic Evolution of Ligands by EXponential enrichment (SELEX) exploits vast combinatorial nucleic acid libraries to identify short, nonimmunogenic single-stranded DNA molecules (aptamers) capable of recognizing specific targets based on their 3D structures and molecular interactions. While SELEX has previously been applied to identify aptamers that bind specific cell types or gain cellular uptake, selection of aptamers capable of carrying cargos to specific subcellular compartments is challenging. Here, we describe peroxidase proximity selection (PPS), a generalizable subcellular SELEX approach. We implement local expression of engineered ascorbate peroxidase APEX2 to biotinylate naked DNA aptamers capable of gaining access to the cytoplasm of living cells without assistance. We discovered DNA aptamers that are preferentially taken up into endosomes by macropinocytosis, with a fraction apparently accessing APEX2 in the cytoplasm. One of these selected aptamers is capable of endosomal delivery of an IgG antibody.

**Significance Statement**

Natural viroids are infectious naked nucleic acids that gain access to subcellular compartments in eukaryotic cells. We are intrigued with the challenge of using in vitro selection to identify DNA aptamers capable of such intracellular homing. Here, we describe a strategy based on proximity biotinylation where vast random libraries of single-stranded DNA oligonucleotides are subjected to selection for access to a biotinylating enzyme engineered to be expressed in the target compartment of interest.

## Introduction

Nanoscale solutions for subcellular targeting of antibodies, small-molecule drugs, and nucleic acids are currently limited. Successful identification and characterization of subcellular compartment-targeting molecules has significant implications for both cell biology and medical therapeutics applications. However, no generalizable methods currently exist to effectively identify tools capable of driving subcellular delivery of molecular “cargo.”

Many existing in vivo and in vitro solutions employ viruses or toxic lipid formulations that distribute payloads across the entire volume of cells after crossing the lipid membrane, decreasing efficacy and allowing for intracellular off-target effects (1). In vivo delivery of CRISPR-Cas9 technologies, for example, is significantly challenged by barriers to cellular uptake (2). Viral platforms are limited by packaging size, have the potential to induce immune response during in vivo application, and cannot address intracellular targeting needs. Other competing engineered approaches including cell-penetrating peptides, lipoplexes, and nanoparticle formulation delivery also have limited subcellular targeting capacity (3). Delivery of oligonucleotide therapies such as antisense and splice-switching oligonucleotides or siRNA duplexes is likewise complicated by both uptake and intracellular trafficking issues (4, 5). Furthermore, small-molecule drugs often rely on diffusion to reach their subcellular sites of action. Without tools to enhance targeting, these drugs require high or repeated dosage (6). Even after drug uptake, the agent still must navigate endosomal escape, diffusion through the viscous cytosolic fluid, and translocation across membrane-bound organelles. Subcellular targeting is especially important for drugs with variable activity depending on intracellular localization (7).

Despite the evident need for tools to improve specific delivery, most approaches for intracellular targeting have historically relied on passive targeting by formulations of liposomes or polymers. We envision here that recent advances in proximity biotinylation technology may be applicable in the search for reagents capable of colocalizing with subcellular compartment-specific enzymes. We further imagine proximity biotinylation as a tactic to engage the power of combinatorial selections of aptamers from vast libraries rather than engineered design.

Proximity biotinylation is a well-established and powerful method for the identification of protein–protein interactions and for mapping subcellular localization of proteins and RNAs with nanometer-scale resolution (8, 9). Biotin ligases BioID and TurboID generate highly reactive biotin-AMP intermediates that label primary amines or other strong nucleophiles present in lysines and N-termini of nearby proteins (10–14), but suitable reactive groups are not available on nucleic acids. Dual RNA and protein proximity labeling is made possible by the engineered ascorbate peroxidases APEX and APEX2, which requires exogenous H_2_O_2_ and biotin tyramide (BT) in cell culture media (9, 15–17). However, we have shown that peroxidase-catalyzed BT phenoxy radicals do not directly biotinylate DNA oligonucleotide substrates. We previously reported that 5′ terminus conjugation to fluorescein enables BT radical biotinylation of DNA oligonucleotides (18). This approach enables proximity biotinylation and streptavidin capture of exogenous single-stranded DNAs in the presence of BT, H_2_O_2_, and APEX2.

APEX2 has been specifically and efficiently expressed in at least nine different subcellular locations including the cytosol, nucleus, mitochondria, and endoplasmic reticulum (15). We hypothesized that proximity biotinylation by localized APEX2 expression in HEK293T cells could be applied to identify single-stranded DNAs (aptamers) capable of cell penetration and homing to APEX2. This approach generalized our prior strategy applied to the selection of karyophilic aptamers wherein an endogenous compartment-specific enzyme, DNA ligase, was required (19).

Here, we apply peroxidase proximity selection (PPS) as a SELEX reward strategy with combinatorial libraries of single-stranded DNA oligonucleotides to identify aptamers capable of localizing to a subcellular region of interest. We demonstrate the principle of PPS with cytosol-targeted APEX2 expression in HEK293T cell culture to select DNA aptamers capable of cytosolic delivery to access biotinylation by APEX2 fused to a nuclear export signal (APEX2-NES).

## Results

We sought to use APEX2-NES-expressing HEK293T cells (15) to select from a library of ∼100 trillion candidate 5′-FAM-conjugated DNA aptamers those capable of gaining access to the cytoplasm. We first confirmed that APEX2-NES is stably expressed, and its activity is specifically localized to cytoplasm (Fig. S1A–C). These cells were then used to produce whole-cell lysates to monitor APEX2 activity. APEX2-dependent biotinylation and streptavidin-dependent gel shift of 5′-FAM-modified library DNA 1 (Table S1) in the presence of APEX2-NES cell lysates confirmed our previous observation (18) that BT radicals are capable of biotinylating 5′-FAM. This enabled magnetic streptavidin bead capture to reward aptamers that achieve proximity biotinylation (Fig. S1C). Biotinylation is observed in whole-cell lysates, but not in overnight cell culture media from the same cells, demonstrating that soluble APEX2 activity is undetectable (Fig. S1D). We therefore employed APEX2-NES-expressing HEK239T cells for studies of PPS using a 5′-FAM-modified DNA library.

**Fig. 1.**
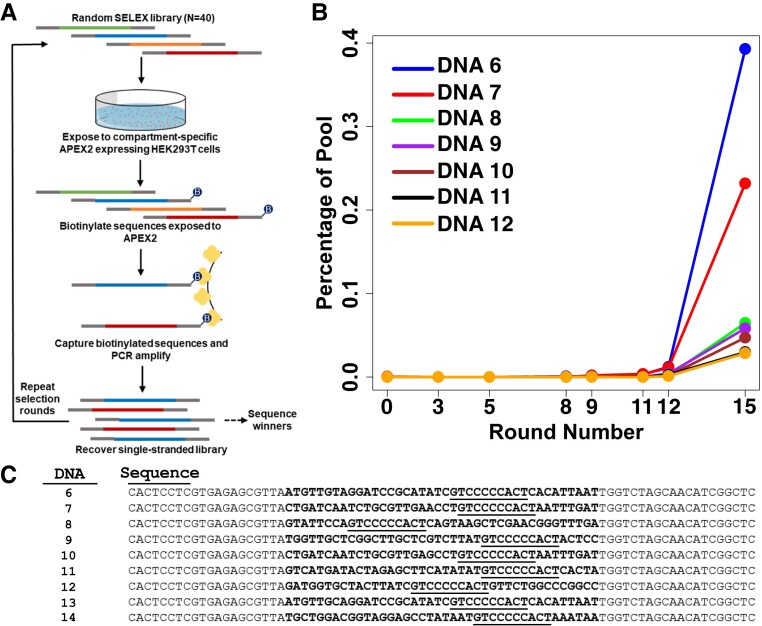
PPS strategy and selection results. A) The naïve library aptamer library consists of a random region (40 nt) flanked by constant sequences (20 nt each) and is exposed in culture media to cells expressing APEX2 targeted to a subcellular compartment of interest. Aptamers colocalizing with APEX2 are biotinylated on a 5′-terminal fluorescein residue, enabling capture on streptavidin magnetic beads and amplification by PCR. B) Fifteen rounds of selection on cells expressing cytosolic APEX2 (APEX2-NES) were performed. The top seven sequences by percentage of library population in round 15 are shown to represent library enrichment. C) Selected aptamer candidates from round 15. The 10-nt motif GTCCCCCACT is underlined in each sequence. Sequences derived from random regions are in bold.

The 80-nt oligonucleotide library DNA 1 consists of an internal 40-nt random sequence flanked by two constant primer binding sequences enabling PCR amplification. Deep sequencing confirmed consistent distribution of all bases at each position in the random region (Fig. S2A). This synthetic library was first used as a PCR template to produce an untrained single-stranded DNA library such that all molecules had 5′-FAM conjugation and locked nucleic acid (LNA) incorporation at three positions in the forward primer homology region. These three positions were chosen arbitrarily near the 5′ terminus of the molecule to protect internalized oligonucleotides from nuclease degradation while having minimal impact on folded structure. LNA modifications were removed from selected aptamers before screening for function. This unselected, 5′-FAM-conjugated, and LNA-protected combinatorial library of ∼3 × 10^14^ DNAs was prepared in fresh cell culture media containing BT and added to cells for 30 min. H_2_O_2_ was then added briefly to activate APEX2, thereby biotinylating DNA aptamers in proximity, presumably by having gained cytoplasmic access. Cells were then thoroughly washed, and total nucleic acids were isolated for streptavidin magnetic bead capture of biotinylated aptamers. Following thorough washing, bead-captured aptamers were used as a template for direct PCR amplification and single strands were then obtained by denaturing gel purification. This process constituted a single selection round (Fig. 1A). Rounds were repeated 15 times with decreasing amounts of library input used beginning at round 2 and again at round 9. Progress at each round was monitored by qPCR, including a BT-free mock selection at round 9 and an APEX2-free mock selection at round 15 to demonstrate that library enrichment depended on the presence of each reagent (Fig. S2B–D) (20). qPCR results indicated recovery above background at each round, though exponential enrichment was not achieved over these cycles.

Aptamers from eight of the selection rounds, including naïve library (“round 0”), were prepared for deep sequencing. No individual aptamer clone was enriched above 0.4% of the final round (Fig. 1B), and deep sequencing and MEME motif analysis (21) revealed that the 10-nt motif GTCCCCCACT (*e*-value = 0.0042; statistically significant) was present in 10% of all oligonucleotides in the round 15 pool. Corresponding library diversity analysis of round 15 found that ∼10% of sequences in the pool were enriched with at least one copy, while 90% of the pool remained singletons (Fig. S2E). Strikingly, of the top 40 sequences in selection round 15, 39 included this full motif and 1 contained the truncated motif GTCCC (Table S2). Many of the most enriched sequences had edit distances of 5 or less from other candidate aptamers in the pool. We therefore selected for study 9 representative aptamers (DNAs 6–14; Table S1) from among the top 40 most enriched sequences (Fig. 1C).

### Aptamer candidate characterization

**Fig. 2.**
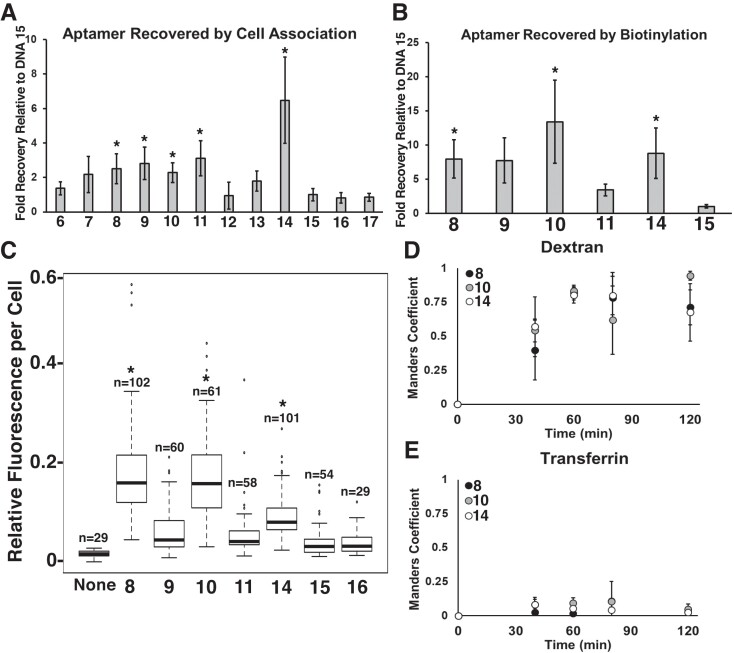
Assays of cell association reveal three best-performing aptamers. A) Results of an assay monitoring association and protection from washing are shown for nine selected aptamer candidates (DNAs 6–14) and negative controls (DNAs 15–17). B) Assay of in vivo biotinylation in the presence of BT and H_2_O_2_ reveals that four of the five candidates selected in A) are reproducibly biotinylated and captured on streptavidin magnetic beads. C) Quantification of relative fluorescence per cell observed in cells after treatment with candidate aptamers selected from A) for the number of cells (*n*) quantified. D, E) Mander's coefficients of colocalization between fluorescent aptamers and fluorescent markers of macropinocytosis (dextran) and clathrin-mediated endocytosis (transferrin). Asterisk indicates *P* < 0.05.

All aptamer candidates and negative controls were synthesized with 5′ FAM conjugation unless otherwise specified. We first tested the nine selected candidates (DNAs 6–14) by a cell association assay comparing these aptamers with three negative control sequences (DNAs 15–17). Negative controls were selected by identifying sequences present in the naïve aptamer library but not found in the final round which therefore must have been selected out of the random pool due to lack of activity. Aptamers were exposed to HEK293T cells lacking APEX2 for 1 h before stringent washing with the goal that any noninternalized oligonucleotides would be dissociated and discarded. qPCR analysis of recovered DNA from cells revealed that five of the nine candidates associated with cells to a greater degree than negative control molecules, interpreted as evidence of cell internalization and protection from washing (Fig. 2A).

These five candidates (DNAs 8, 9, 10, 11, and 14) were further tested for their ability to enter cells and achieve proximity biotinylation by APEX2-NES, presumably in the cytoplasm. Aptamers were prepared in fresh media and incubated with APEX2-NES-expressing HEK293T cells for 1 h in the presence of BT. qPCR was again used to assess DNA recovery following APEX2-NES activation by H_2_O_2_ and streptavidin bead capture of biotinylated DNAs (Fig. 2B). Four aptamers were biotinylated and captured on streptavidin beads to a greater extent than negative control molecule DNA 15. These same aptamers could not be biotinylated and captured from HEK293T cells lacking APEX2-NES (Fig. S3A). To exclude the possibility that biotinylation occurred in media by APEX2 released from lysed cells, excess tyrosine was added to media during incubation as a radical quenching agent with best-performing aptamer DNA 8 and negative control DNA 15 (22). Tyrosine in up to 12,500-fold molar excess relative to aptamer did not interfere with biotinylation of DNA 10 and subsequent bead capture (Fig. S3B). We also demonstrated that selected aptamers were not identified due to exceptional biotinylation properties. Aptamers and negative controls were exposed to APEX2-NES HEK293T cell lysates in the presence of exogenous BT and H_2_O_2_. Following in vitro biotinylation in lysates, DNAs were isolated and captured on magnetic streptavidin beads for subsequent qPCR quantification. This analysis revealed that no aptamers were biotinylated more efficiently than negative control DNA 15 (Fig. S3C). This result suggests that selected aptamers were not captured on beads due to exceptional activities as BT radical targets. We also showed that aptamers were not enriched during selection by direct streptavidin binding (Fig. S3D) or by endosomal release into cytosol upon H_2_O_2_ treatment (Fig. S3E).

Aptamer internalization was further examined by confocal microscopy using Alexa Fluor 647 labeling. We first quantified relative aptamer fluorescence to confirm that aptamers were similarly labeled by Alexa Fluor 647 (Fig. S3F). Fluorescently labeled DNAs 8, 10, and 14 were detected in counter-stained HEK293T cells. Staining by DNAs 9 and 11 and negative control DNAs 15 and 16 was not observed (Figs. 2C and S4). Interestingly, uptake of DNAs 8, 10, and 14 was observed in distinct puncta rather than throughout the cytoplasm. We interpret this as evidence that aptamers selected for cytoplasmic access are most concentrated in endosomes, and presumably, the minority of molecules that reach the cytoplasm for proximity biotinylation are not detected by microscopy.

**Fig. 3.**
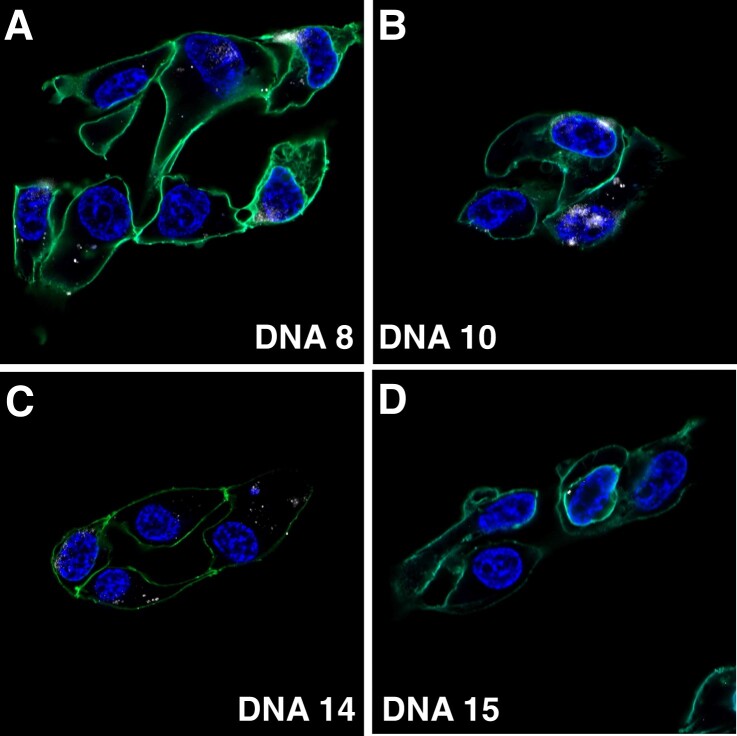
Confocal microscopy assessment of aptamer uptake in chloroquine-treated HEK293T cells. HEK293T cells pretreated with 10 µm chloroquine for 4 h show enhanced size and intensity of fluorescent aptamer puncta for A) DNA 8, B) DNA 10, and C) DNA 14. D) Uptake of negative control DNA 15 is not detected under these chloroquine treatment conditions. Images collected at 100× magnification.

To determine types of endosomes containing aptamers, we exposed cells to fluorescent markers specific for macropinocytosis or clathrin-specific uptake and monitored overlap with aptamer fluorescence. Macropinocytosis-specific fluorescent dextran was found to significantly overlap with all three aptamers in a time-dependent manner as quantified by the Mander coefficient of fluorescence correlation (Fig. 2D; representative image Fig. S5). In contrast, little correlation was observed with clathrin-specific transferrin puncta (Fig. 2E; representative image Fig. S6). Chloroquine, a lysosomotropic agent that interferes with lysosomal degradation by raising endosome pH (23), was used to disrupt lysosomal activity in an attempt to enhance aptamer visualization. Indeed, chloroquine treatment before and throughout aptamer incubation increased the number, size, and intensity of visible aptamer puncta (Fig. 3). Aptamer staining was markedly more perinuclear after chloroquine treatment. Uptake of DNA 9 and DNA 11 was not detectable in the presence of chloroquine.

Best-performing aptamers DNA 8, DNA 10, and DNA 14 were next challenged by exposure to media and cell lysate nucleases to confirm that they had not been inadvertently selected for unusual nuclease resistance. These three aptamers were not more stable in fresh or conditioned cell culture media than negative control molecule DNA 15 after 3 h of incubation at 37°C (Fig. S7).

Secondary structures of DNAs 8, 10, and 14 were predicted using mfold (24). Primary features of predicted structures were validated using mung bean nuclease protection and KMnO_4_ oxidation assays, both sensitive to base pairing (Figs. S8A and B, S9A and B, and S10A and B). We measured melting temperatures of folded aptamers, finding that DNA 8 (*T*_m_: 56°C), DNA 10 (*T*_m_: 41°C), and DNA 14 (*T*_m_: 46°C) are all expected to be stably folded in cell culture conditions (Figs. S8C and D, S9C and D, and S10C and D).

### Protein delivery

**Fig. 4.**
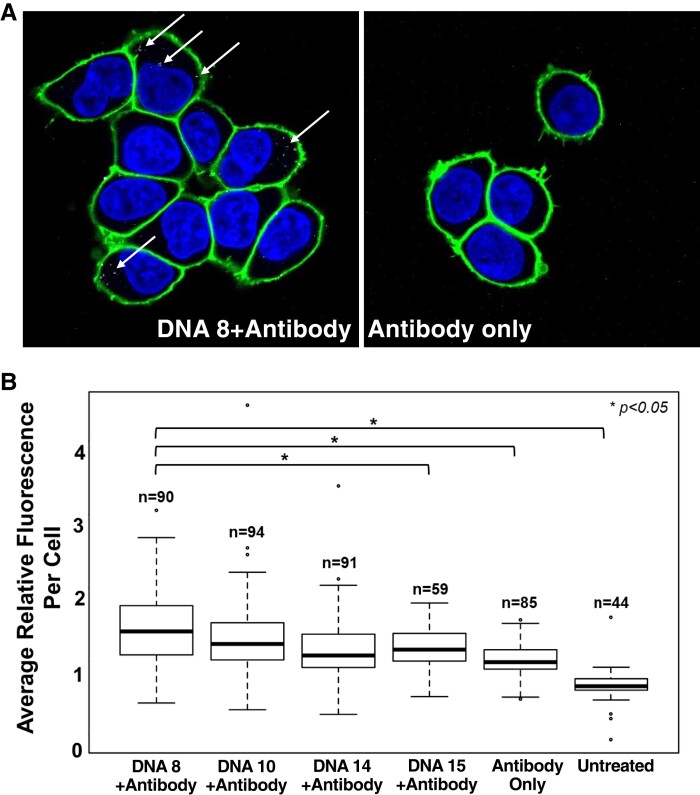
DNA 8 enables internalization of an antidigoxigenin antibody. A) Confocal microscopy demonstrating cell uptake of fluorescently labeled antidigoxigenin antibody bound to digoxigenin-modified DNA 8. Antibody alone is not internalized. Green: CellBrite membrane stain. Blue: DAPI. Images collected at 100× magnification. B) Quantification of relative antidigoxigenin antibody fluorescence per cell. Only DNA 8 is capable of antibody cargo delivery. *n*, number of cells quantified per condition.

After confirming uptake of DNA 8, DNA 10, and DNA 14 by cell association assay, APEX2-NES biotinylation assay, and confocal microscopy, we tested these three aptamers as candidates for protein cargo delivery. Each sequence was synthesized with a 5′ terminal digoxigenin modification and incubated with a fluorescently labeled antidigoxigenin antibody (Fig. S11) for noncovalent complex formation before incubation on HEK293T cells. Aptamer–antibody conjugates were assembled with aptamer in excess to ensure that any observed fluorescent signal reflected conjugated protein. Significant fluorescent antibody internalization was detected by confocal microscopy for DNA 8–antibody conjugates (Fig. 4A). Fluorescent antibody delivery by DNA 10 and DNA 14 was not statistically significant compared with signal observed for aptamer conjugated to negative control DNA 15 or unconjugated antibody alone (Fig. 4B). We observed that delivered antibodies were only visible in a fraction of cells. However, cells that did take up antibody displayed multiple puncta per cell. This result indicates that selected DNA 8 (∼26 kDa) can deliver a ∼150 kDa protein for cell uptake.

### Characterization of the conserved 10-nt motif

We next explored the role of the conserved 10-nt motif GTCCCCCACT present in ∼10% of sequences in the final selection round. We chose DNA 8 for this analysis given its unique antibody delivery capabilities. DNA 8 derivative DNA 18 was generated with the shuffled motif CCACGTCCTC substituted for GTCCCCCACT in an otherwise identical sequence. Negative control DNA 15, which did not contain the 10-nt motif of interest, was synthesized as DNA 19 with the GTCCCCCACT motif inserted into the loop of a predicted stem–loop structure as is the case in DNA 8 (Fig. S12A–E). Rearrangement of the 10-nt motif to create DNA 18 eliminated cell association (Fig. S12F). Substitution of GTCCCCCACT into DNA 15 to generate DNA 19 did not endow cellular uptake to the inactive oligonucleotide (Fig. S12F). This study suggests that the GTCCCCCACT motif is required for cell uptake but is not sufficient to confer activity and is, perhaps, dependent on folded aptamer structural context.

### Secondary structure features of DNA 8

Given that the isolated GTCCCCCACT motif of DNA 8 is not sufficient for cell internalization, we next explored minimal secondary structure features required in addition to this sequence. We generated two truncated versions of DNA 8. DNA 24 was created by deleting 5 nucleotides predicted to be unstructured at the 5′ terminus of DNA 8 along with 24 nucleotides participating in a predicted 3′ hairpin loop (Fig. S13A). DNA 25 was created by truncating a further seven nucleotides from the 5′ and 3′ termini of DNA 24, disrupting the 11-nt stem predicted to form between these regions (Fig. S13A). Whereas the removal of the predicted 3′ hairpin loop of DNA 8 did not inhibit endosomal uptake, disruption of the internal stem formed by nucleotides 6–16 and 45–55 inhibited uptake (Fig. S13B).

### Cell type specificity of DNA 8

We tested cell association for seven cultured cell lines in addition to the HEK293T selection line to assess cell specificity of DNA 8. Tested cell lines were as follows: HeLa, cervical adenocarcinoma; U2OS, osteosarcoma; SH-SY5Y, neuroblastoma; MCF7, breast adenocarcinoma; MDAH, ovarian carcinoma; PANC1, pancreatic carcinoma; and A549, lung carcinoma. DNA 8 was not detectably internalized (versus negative control DNA 15) in five of seven cell lines; U2OS and MDAH cell cultures demonstrated sequence-specific activity (Fig. S14A), but to a lower extent than for HEK293T cells. Given our finding that DNA 8 uptake is correlated with uptake of macropinocytosis-specific fluorescent dextran in HEK293T cells (Fig. 2D and E), we predicted that relative rates of macropinocytosis between the studied cell lines could partially explain differential aptamer internalization. Quantifying relative amounts of fluorescent 70 kDa dextran internalization revealed that the cell lines with the highest rates of macropinocytosis were HEK293T, U2OS, MDAH, and PANC1 (Figs. S14B and S15). Among these four, only PANC1 cells did not specifically internalize DNA 8 (Fig. S14).

### Proteomic analysis of aptamer–cell interactions

**Fig. 5.**
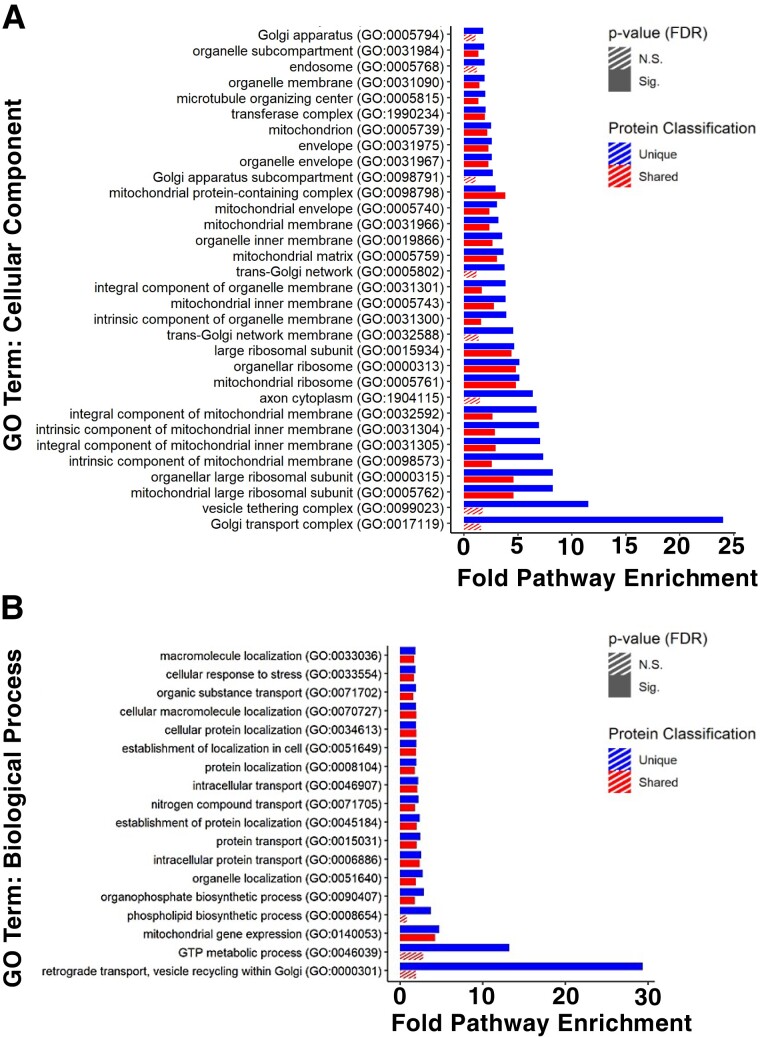
Proteomic analysis of aptamer–cell interactions of DNA 8. GO overrepresentation analysis of proteins consistently interacting with DNA 8 but not with negative control DNA 15 reveals multiple highly enriched and statistically significant pathways among both A) cellular component and B) biological process GO terms. For each enriched and significant GO term overrepresented among unique interactions, proteins interacting with both DNA 8 and DNA 15 are compared and statistical significance is assessed.

DNA 8, DNA 10, and DNA 14 were each used for cross-linking analysis with the goal of illuminating cell internalization and trafficking mechanisms. 3′ biotin conjugates of each aptamer and negative control DNA 15 were incubated with cells, cross-linked with formaldehyde, and captured on streptavidin magnetic beads following cell lysis. Biological replicates of aptamer–cell and negative control–cell interactions were employed. Proteins represented by peptides cross-linked to all aptamer replicates, but excluded from all negative control replicates, were analyzed in Gene Ontology (GO) enrichment analysis to identify overrepresented pathways specific to subcellularly targeted aptamers. Cellular component GO overrepresentation analysis revealed highly enriched and statistically significant associations of all three selected aptamers with endosomal proteins, Golgi apparatus transport complexes, and vesicle tethering complexes (Figs. 5A and S16A and B). Notably, each of these GO terms was statistically significant only for proteins uniquely associated with each aptamer. Biological process GO analysis additionally identified enrichment for retrograde Golgi transport processes for DNA 8 (Fig. 5B). Interestingly, cellular component analysis also identified statistically significant enrichment of terms associated with mitochondrial membrane proteins for DNA 8 (Figs. 5B and S16A and B). Finally, similar enrichment was found for proteins cross-linked by DNA 8 and negative control DNA 15 for cellular component GO terms associated with cell surface and cellular periphery (Fig. S17).

## Discussion

Here, we describe the first example of an approach that we term PPS to identify DNA aptamers capable of localizing to subcellular compartments. FAM-conjugated DNA aptamers are amenable to peroxidase-meditated biotinylation and were successfully selected for their ability to localize within the labeling radius of a cytosolic form of APEX2 in HEK293T cells. This capability was suggested by multiple independent methods. Notably, DNA 8 directed intracellular delivery of a bound IgG antibody approximately six times its size.

Fifteen rounds of PPS yielded several aptamer sequences containing a conserved 10-nt motif GTCCCCCACT. We screened these candidates in a simple assay testing cell association and protection from washing. Aptamer internalization into HEK293T cells lacking APEX2 expression prevented their loss after washing, as monitored by qPCR (Fig. 2A). This result demonstrates that aptamer internalization does not require APEX2-NES, but rather results from unique aptamer interactions with HEK293T cells. Screening candidates by cell association assays resulted in a narrowed list of five aptamer sequences that were then tested for their ability to be biotinylated by APEX2-NES in HEK293T cells as a measure of cytosolic APEX2 proximity. Best-performing candidates incubated with cells together with BT and H_2_O_2_ were biotinylated and detected by streptavidin bead and capture 10- to 15-fold more efficiently than negative control sequences (Fig. 2B). A 12,500-fold molar excess of BT-quenching tyrosine in cell culture media did not inhibit bead capture, suggesting that aptamer biotinylation occurs intracellularly (Fig. S3B). It is unlikely that aptamers were selected for intrinsic biotinylation propensity because these sequences demonstrated equal reactivity with BT radicals in vitro (Fig. S3C).

Following aptamer internalization and biotinylation assays, we confirmed cellular aptamer uptake by confocal microscopy. Fluorescent aptamer internalization was observed in distinct puncta within cell membranes, consistent with uptake and concentration in endosomes prior to cytoplasmic delivery. We assumed that this endosomal localization leads to cytoplasmic delivery for PPS, though cytoplasmic aptamer signal is below fluorescence detection levels. Endosomal delivery was markedly enhanced by pretreatment of cells with chloroquine (Fig. 3), which alters lysosomal pH, preventing the activation of acid-dependent enzymes that typically degrade lysosome-trafficked proteins and nucleic acids. We interpret these effects as evidence that a detectable fraction of aptamers are trafficked through vesicles to lysosomes. The endosomal aptamer signal is likely due to their higher concentration in vesicles relative to cytosol. Our examination of nuclease resistance suggests that aptamers do not accumulate simply on the basis of enhanced nuclease resistance (Fig. S8). We therefore conclude that aptamer uptake required for biotinylation must be occurring through a mechanism allowing a fraction of aptamers in endosomes to escape into cytosol enabling PPS before lysosomal degradation. Evidence of aptamer-specific endosomal protein interactions suggests that uptake and endosomal escape could be facilitated by either protection from degradation after binding or by cotrafficking with proteins out of endosomes. This endosome-released fraction of aptamers is detectable by APEX2 proximity biotinylation, but not by fluorescent imaging.

Oligonucleotide trafficking could occur by several endocytosis pathways. We studied this by examining aptamer colocalization with fluorescent pathway-specific markers. We found that aptamers exclusively colocalized with fluorescein-labeled 70 kDa dextran in HEK293T cells but were excluded from vesicles marked by clathrin-specific transferrin (Fig. 2D and E). Vesicular colocalization with 70 kDa dextran, an established marker of macropinocytosis, suggests that selected aptamers are first endocytosed by macropinocytosis and then intracellularly trafficked by a currently unknown mechanism that is not activated by negative control oligonucleotides. Future work will focus on uncovering mechanistic details.

Interestingly, the 10-nt motif GTCCCCCACT found in 10% of the round 15 aptamers was necessary but not sufficient for aptamer internalization. We demonstrated that this motif is required for cell internalization of 80-nt DNA 8 and is part of a core 50-nt component of the aptamer that is also required for activity (Figs. S12 and S13). We hypothesize that this 10-nt motif may interact with a cell surface or endosomal binding partner to promote cellular uptake or endosomal escape into cytoplasm.

Proteomic analysis of aptamer-cross-linked proteins upon formaldehyde treatment identified statistically significant enrichment among endocytosis and endosome trafficking-related proteins for DNA 8. Though no single-cell surface or endosomal protein target was identified in this study, these results support our observations of internalization-specific aptamer activity compared with a negative control DNA molecule. Twenty-four proteins uniquely captured by biotinylated DNA 8 were identified among early and late endosomes, multivesicular bodies, lysosomes, and plasma membrane. The mechanism of endosomal escape may require the highly enriched 10-nt GTCCCCCACT motif through its interaction with a specific protein, but further work will be required to identify such a partner.

DNA 8 was shown to drive intracellular delivery of a fluorophore-labeled antidigoxigenin antibody. This result demonstrates the potential of PPS-selected aptamers to deliver molecular cargos many times their size. Though DNA 8-mediated antibody delivery was observed in only a fraction of cells, these cells displaying aptamer internalized showed multiple fluorescent puncta in the cytoplasm (Fig. 3). Postselection conjugation of large cargos may interfere with aptamer folding. Future selections may be improved if aptamer libraries are preconjugated with intended cargos so that selected pressures are in the context of such modifications. This approach may be especially useful when delivering protein cargos.

This work provides the first example of peroxidase proximity aptamer selection, an approach theoretically applicable to a wide range of subcellular compartments and cell types. This approach suggests future selections of aptamers targeting increasingly challenging subcellular compartments including nuclei, mitochondria, and endoplasmic reticulum. Beyond this BT- and H_2_O_2_-dependent PPS method, we are pursuing additional proximity biotinylation strategies enabling selection of cell-penetrating aptamers.

## Materials and methods

### Construction of naïve DNA library for aptamer selection

DNA oligonucleotides were synthesized by Integrated DNA Technologies. Abbreviations in oligonucleotide sequences “/56-FAM/,” “+,” “N,” and “/iSp9/” indicate 5′ fluorescein, LNA-modified bases, random bases, and internal 9-atom spacers, respectively. Library DNA 1 (Table S1) was reconstituted in water to 1 μm. Primers DNA 2 and DNA 3 (Table S1) were reconstituted in water to 5 μm. The naïve DNA library was constructed by PCR using the following reagent volumes: 200 μL 10× Taq polymerase buffer, 200 μL 10× BSA, 160 μL 50 mm MgCl_2_, 160 μL 2.5 mm dNTPs, 80 μL 1 μm library DNA 1, 200 μL 5 μm forward primer 2, 200 μL 5 μm reverse primer 3, 760 μL water, and 40 μL Taq polymerase (Invitrogen # 10342020). Reagents were mixed, aliquoted into 100 μL volumes in PCR tubes, and subjected to thermal cycling with a variable number of repeats using the following protocol: 94°C, 30 s (94°C, 30 s; 54°C, 35 s; and 72°C, 30 s). DNA was thermocycled only three times before round 1 in order to incorporate chemical modifications while minimizing PCR bias and maintaining library diversity.

Following PCR, reactions were pooled and precipitated with EtOH and NaOAc as described above. Nucleic acids were resuspended in 50 μL water and combined with 50 μL formamide. This mixture was heated at 90°C for 5 min before loading into a 10% denaturing polyacrylamide gel (7.5 m urea, 19:1 acrylamide:bisacrylamide) and subjected to electrophoresis for 2 h at 600 V (26 V/cm). DNA bands were visualized by UV shadowing, and desired 80-nt bands were excised from gel by a clean razor blade. The band was diced into small cubes, and DNA was eluted at 55°C overnight in 500 μL 2× PK buffer [100 mm Tris-Cl (pH 7.5), 200 mm NaCl, 2 mm EDTA, and 1% SDS] with agitation. The supernatant with eluted DNA was then collected and combined with an equal volume of phenol:chloroform. The upper aqueous phase was transferred, and DNA was precipitated from ethanol as described above.

### DNA aptamer selection in cell culture

Naïve DNA library was prepared as described above. Five hundred picomoles (round 1), 250 pmol (rounds 2–8), or 200 pmol (rounds 9 and beyond) was prepared in 200 μL volumes with a final concentration of 1× PBS containing 1 mm MgCl_2_ and heated at 90°C for 5 min to denature single strands and then placed in ice water to snap cool. The library was then added to 2,800 μL DMEM medium containing 10% FBS (R&D Systems, S11195H) and pen/strep antibiotics (Gibco # 15140122) with 3 μL of 500 mm BT in DMSO for a final concentration of 500 μm BT. Medium was aspirated from a 10-cm dish of 80% confluent HEK293T APEX2-NES cells and replaced with 3 mL solution of media containing BT and aptamer library. Cells with aptamer library were incubated for 30 min at 37°C with gentle mixing every 10 min. Following this, incubation medium was aspirated and cells were washed once in fresh media before the addition of 3 mL fresh media containing 100 mm H_2_O_2_. Cells were incubated at 37°C for 1 min with peroxide, after which medium was again aspirated and cells were washed twice for 10 s in room-temperature PBS containing 0.5 m NaCl. Two 30-s washing steps were then performed with room-temperature PBS, and cells were scraped into 1 mL PBS and transferred to a 1.7-mL microcentrifuge tube. Cells were pelleted by a gentle spin at 500 × *g* for 5 min and resuspended in 400 μL PBS.

Cells in PBS were held at 90°C for 5 min with intermittent vortex treatment to lyse. Four hundred microliters of phenol:chloroform solution was combined with lysates followed by thorough vortex mixing, and samples were subjected to centrifugation at 16,000 × *g* for 5 min or until phases were well separated. Nucleic acids in the upper aqueous layer were precipitated as described above and resuspended into 50 μL water.

M-270 Streptavidin Dynabeads beads (Invitrogen # 65305) were used to capture biotinylated oligonucleotides. Two hundred microliters (rounds 1–8) or 100 μL (rounds 9+) of stock bead solution was transferred to a microcentrifuge tube and combined with 1 mL 1× Bind and Wash (B&W) buffer (10 mm Tris-HCl, pH 7.5, 1 mm EDTA, 2 m NaCl, and 0.1% Tween-20). Endogenously biotinylated RNA isolated by total nucleic acid precipitation is expected to compete with biotinylated exogenous library for bead binding, which could hinder library capture if bead capacity is limiting. M-270 bead capture capacity was therefore used in significant excess of aptamer library input to ensure complete capture of any scare biotinylated DNA. Beads were placed on a magnetic stand for 1 min to separate from buffer and supernatant was discarded, followed by two washes in 200 μL 1× B&W buffer. Beads were finally resuspended in 50 μL 2× B&W buffer and combined with 50 μL isolated nucleic acids from the selection step with thorough mixing by pipetting and incubated together at room temperature for 1 h with intermittent mixing as beads settled. Following bead capture, beads were again placed on a magnet stand and unbound DNAs were discarded. Beads were then extensively washed to remove any remaining unbound DNAs by the following steps: twice with 200 μL 1× B&W buffer (wash A), once with 200 μL 0.1 m NaOH (wash B), twice with wash A, twice with wash B, twice with wash A, and once in water. Well-washed beads were resuspended in 50 μL water.

M-270 Dynabeads with bound oligonucleotides can serve as DNA templates in place of isolated DNA during PCR. Therefore, beads with captured oligonucleotides were used directly as templates for analytical PCR to determine an optimal number of PCR cycles to be used in a large-scale PCR. Large-scale PCR products were precipitated from ethanol, and desired strands were isolated from denaturing gels as described above.

### Aptamer sequencing

Recovered DNA libraries from rounds of interest were used to regenerate PCR products for high-throughput sequencing. Seven of the eight sequenced rounds were chosen to represent selection progress with a focus on later rounds (0, 3, 5, 8, 11, 12, and 15). Round 9 was selected for sequencing due to our decision to perform a parallel BT-free negative control (Fig. S2C) at this round. Libraries were amplified for the number of cycles determined by analytical PCR in each selection round using unmodified primers 3 and 4 (Table S1). PCR reactions were as follows: 5 μL 10× Taq polymerase buffer, 5 μL 10× 1 mg/mL BSA, 4 μL 50 mm MgCl_2_, 4 μL 2.5 mm dNTPs, 3 μL library, 5 μL 5 μm forward primer DNA 4, 5 μL 5 μm reverse primer DNA 5, 20 μL water, and 1 μL Taq polymerase. PCR product size and quality were assessed by electrophoresis in 10% native polyacrylamide gel (19:1 acrylamide:bisacrylamide) before proceeding.

PCR products were then purified using a MinElute Purification Kit (Qiagen # 28004), and eluted DNA was quantified using a Qubit HS Duplex DNA Quantification Kit (Invitrogen # Q32851). Ten nanograms of duplex DNA was used as input for the NEBNext Ultra II DNA Library Prep Kit (NEB # E7645S) using associated NEBNext Multiplex primers. After preparing per the manufacturer’s specifications, samples were analyzed by high-throughput paired-end 150 cycle sequencing on an Illumina MiSeq instrument. Sequencing results were then analyzed using AptaSuite software.

Following deep sequencing analysis of the first round, we identified three randomly selected sequences that were present in the naïve unselected library and absent from the final round 15 library. These sequences, which were selected out of the pool, served as negative controls DNA 15, DNA 16, and DNA 17.

### Cell association and biotinylation assays

HEK293T cells were grown to ∼80% confluency in 12-well plates (Corning # 3513) overnight for use the following day. One hundred picomoles per well of candidate oligonucleotides was prepared in 100 μL PBS containing 1 mm MgCl_2_ with heating and snap cooling as described above. One hundred microliter solutions were added to 900 μL fresh media to create 1 mL media with aptamer per well. Cells were incubated in media with 5′ FAM-modified aptamers for 1 h with gentle agitation to mix every 10–20 min. Medium was then aspirated from plates, and cells were washed twice with PBS containing 0.5 m NaCl and three times with PBS. Thoroughly washed cells were scraped with 200 μL PBS and collected in microcentrifuge tubes. Lysis by heating and DNA isolation by EtOH precipitation were performed as described above. Recovered cell-associated oligonucleotides were quantified by qPCR using QuantaBio SYBR Green FastMix (QuantaBio # 95071).

Oligonucleotides for biotinylation and bead capture assays were prepared identically to cell association assays, with the exception that 200 pmol aptamer was used. BT was included in media for the duration of the 1-h incubation before cells were washed once with fresh media. Fresh medium with 100 mm H_2_O_2_ (1 mL) was added to washed cells for 1 min before washing again as described in the above aptamer selection protocol. Biotinylated oligonucleotides were also captured on M-270 streptavidin magnetic beads using the same method, and recovery was quantified by qPCR.

### Confocal microscopy

Cells were plated at 50–70% confluency in DMEM containing 10% FBS with antibiotics on glass-bottom dishes and allowed to adhere overnight. Aptamer (250 pmol per well) was prepared in 100 μL PBS with 1 mm MgCl_2_ and heated and snap cooled as described above. Aptamer solutions (100 μL) were added to 900 μL fresh media to create 1 mL solution per well. Cells were incubated in media with aptamers for indicated times. Where indicated, chloroquine (Sigma-Aldrich # C6628) was added to media at 10 μm and cells were incubated for 4 h at 37°C prior to the addition of aptamer with media including chloroquine. Following aptamer uptake, cells were washed with PBS with 0.5 m NaCl and PBS as in cell association assays. Washed cells were fixed on dishes using 3.7% formaldehyde solution in PBS for 15 min at room temperature and washed once with PBS to remove excess formaldehyde. Cells were washed once in PBS to remove excess formaldehyde and stained with DAPI diluted in PBS per manufacturer's recommendation for 5 min. DAPI-stained cells were washed twice with PBS before imaging. For images including CellBrite Green Cytoplasmic Membrane Dye (Biotium # 30021), dye was diluted at 2.5 μL/mL in PBS and cells were stained in this solution at room temperature for 10 min protected from light. Uptake of 5′ Alexa Fluor 647-labeled aptamers was observed by confocal microscopy on a Zeiss LSM 780 microscope and quantified using CellProfiler (25).

Fluorescent 70 kDa dextran (Invitrogen # D1823) was used to track uptake via macropinocytosis. Stock solution was diluted to 250 μg/mL in fresh media for experiments requiring dextran. Fluorescently labeled transferrin (Invitrogen # T2871) was similarly prepared at 25 μg/mL. Incubation times for both pathway markers are as indicated. Cells stained with pathway markers were washed three times in PBS before proceeding to fixation and DAPI staining. Mander's coefficients of pathway marker and aptamer overlap were calculated using the CellProfiler Mander's coefficient module. Correlation quantification was performed across three biological replicates with one image taken per replicate at each indicated time. Dextran and transferrin labeling experiments were performed within a 72-h window, minimizing differences in passage number between experiments. For experiments quantifying relative rates of macropinocytosis among cell lines, cells were grown overnight on glass-bottom dishes and dextran was prepared as described above. Cells were washed once with room-temperature PBS before adding media with supplemented dextran and incubating for 30 min at 37°C. Cells were then washed twice with room-temperature PBS before formaldehyde fixation and DAPI staining. Four fields were collected across two biological replicates per cell line, and CellProfiler was used to quantify observed fluorescent signal.

### Antidigoxigenin antibody delivery

One hundred picomoles of DNAs 20–23 was combined with 10 μL Alexa Fluor 594 antidigoxigenin antibody (Vector Labs # DI-7594.5) and 30 μL fresh media. The mixture was incubated for 30 min at room temperature to allow aptamer–antibody interaction. Binding of antibody to modified oligonucleotides was assessed by gel shift in 10% native polyacrylamide gel with electrophoresis at 27 V/cm in 0.5× TBE.

Antibody-bound oligonucleotide solutions were combined with 950 μL fresh media and added to HEK293T cells grown to ∼60–80% confluency overnight on glass-bottom dishes. Cells were then incubated for 1 h at 37°C before aspirating media and washing twice with PBS. Following washing, cells were fixed in 3% formaldehyde solution in PBS for 15 min at room temperature before washing once more in PBS to remove excess formaldehyde. CellBrite Green and DAPI staining was then performed as described above. Uptake of Alexa Fluor 647-labeled antibody was observed by confocal microscopy on a Zeiss LSM 780 microscope and quantified using CellProfiler with manual tracing of cells.

### Aptamer–protein interaction analysis

HEK293T cells were grown overnight to ∼80% confluency on a 10-cm cell culture dish. 3′ biotinylated oligonucleotides were added to fresh growth media at 1 μm and were added to cells after aspirating overnight media. Oligonucleotides were incubated on cells for 2 h at 37°C before washing 3× with PBS. Washed cells were then fixed in 3.7% formaldehyde in PBS for 20 min at room temperature to cross-link and capture aptamer–protein interactions. Cells were then washed twice in PBS to remove excess formaldehyde and scraped into 500 μL nondenaturing cell lysis buffer (10 mm Tris-HCl, pH 7.5, 10 mm NaCl, and 0.5% IGEPAL) before being subjected to sonication twice for 10 s to lyse. Fifty-microliter M-270 streptavidin magnetic beads prepared by washing as described above were added to whole-cell lysates and incubated at 4°C with end-over-end rotation for 2 h to capture biotinylated oligonucleotides and associated proteins.

Following oligonucleotide binding, beads were captured on a magnetic stand and washed 10 times in lysis buffer to reduce nonspecific protein–bead interactions. After the final wash, beads were resuspended in 50 μL elution buffer (10 mm Tris-HCl, pH 8.0, 10 mm EDTA, 5 mm DTT, and 1% SDS) and held at 65°C overnight to reverse cross-links. Eluted protein concentrations were quantified using the Qubit Protein Quantification Assay kit (Invitrogen # Q33211). Samples were further characterized by electrophoresis in 10% SDS–PAGE gel (Invitrogen # NP0301) for 40 min at 16 V/cm with visualization by Imperial Protein Stain (Thermo # 24615). Remaining protein was snap cooled and stored in elution buffer at −80°C until submission for proteomic analysis.

## Supplementary Material

pgad151_Supplementary_Data

## Data Availability

All relevant data are presented here or in the supplemental material.
