# Supplementary material for: Peroxidase proximity selection to identify aptamers targeting a subcellular location
Source: PNAS Nexus. 2023 May 4;2(5):pgad151. doi: 10.1093/pnasnexus/pgad151 (PMC10210619; doi:10.1093/pnasnexus/pgad151)
Supplement: pgad151_Supplementary_Data [file pgad151_supplementary_data.pdf]

Peroxidase Proximity Selection to Identify Aptamers Targeting a Subcellular Location

Brandon Wilbanks, William Beimers, Maria Dugan, Taylor Weiskittel, LJ Maher III

Table of Contents

1.0 Supplemental Methods
- 1.1 Confirmation of APEX2 Localization
- 1.2 Confirmation of APEX2 Activity
- 1.3 Monitoring APEX2 Activity in Cell Lysates
- 1.4 Thermal Denaturation Analysis
- 1.5 Aptamer Stability in Cell Lysate
- 1.6 Aptamer Stability in Cell Culture Media
- 1.7 Chemical and Enzymatic Secondary Structure Determination
- 1.8 Aptamer-Protein Interaction Analysis

2.0 Supplemental data

1.0 Supplemental Methods

1.1 Confirmation of APEX2 Localization

APEX2-NES cells (the generous gift of Alice Ting) were plated at 50-70% confluency in DMEM (Gibco # 41965039) containing 10% FBS (R&D Systems # S11550H) and 100 U/mL antibiotics (Gibco # 15140148) on glass-bottom dishes (MatTek # P356-1.5-14-C) and allowed to adhere overnight. Adherent cells were fixed on dishes in 3.7% formaldehyde solution (Millipore Sigma # F8775) in PBS (Gibco # 14190250) for 15 min at room temperature and washed once with PBS to remove excess formaldehyde. Following fixation, cells were treated with permeabilization buffer (PBS containing 0.1% Triton-X 100) for 15 min at room temperature and washed once with PBS to remove excess buffer. Permeabilized cells were stained with anti-Flag primary antibody (Cell Signalling Technology # 132025) diluted 1:500 in antibody dilution buffer (PBS containing 10% FBS) for 1 h at room temperature with gentle rocking and washed 5× in PBS for 5 min per wash. Primary antibody was then detected by staining with secondary AlexaFluor 647 anti-rabbit antibody (Invitrogen # A21245) diluted 1:500 in antibody dilution buffer for 1 h at room temperature. Cells were again washed 5X in PBS stained with DAPI (Roche # 10236276001) diluted in PBS for 5 min. DAPI stained cells were washed twice with PBS before imaging. Images were obtained by confocal microscopy on a Zeiss LSM 780 microscope. Quantification of cell fluorescence was performed with CellProfiler.

1.2 Confirmation of APEX2 Activity

Cells were plated at 50-70% confluency in DMEM containing 10% FBS with antibiotics on glass-bottom dishes and allowed to adhere overnight. Biotin tyramide (BT) (ApexBio # A8011, final concentration 500 μM) was added to media and cells were incubated for 30 min at 37°C. Media containing BT was aspirated and cells were washed twice in fresh media to remove excess BT. After washing, media was combined with 12 μL/mL of 83× H_2O_2 solution and added to cells for 1 min. 83× H_2O_2 solution was prepared by diluting H_2O_2 stock (Sigma # H1009) at 10μL/mL into fresh media. Peroxide-treated cells were washed gently twice in PBS and fixed and permeabilized in 100% methanol for 10 min at –20°C. Methanol was aspirated and cells were washed

twice with PBS. Fixed and permeabilized cells were then stained with AlexaFluor 647-labeled streptavidin (Invitrogen # 521374) diluted 1:1000 in PBS for 1 h at room temperature. Following streptavidin staining, cells were washed 3× in PBS and stained with DAPI diluted in PBS for 5 min. DAPI stained cells were washed twice with PBS before imaging.

1.3 Monitoring APEX2 Activity in Cell Lysates

Cells were grown overnight to 70% confluency in DMEM including 10% FBS and antibiotics in 10 cm plates and washed once in PBS. Washed cells were scraped from dishes into 1 mL PBS into 1.7 mL microcentrifuge tubes and pelleted by centrifugation for 5 min at 300 × g. This was followed by resuspension into 500 µL PBS with Complete Mini Protease Inhibitor cocktail (Roche # 118361530014; one tablet/10 mL). Resuspended cells were sonicated 3× for 10 s on intensity 7 with 2-min incubation on ice between each sonication. Lysed cells were then spun for 15 min at 10,000 × g to pellet debris and supernatants were collected and stored on ice for immediate use. 75 µL of lysate supernatant was combined with 100 pmol fluorescein-conjugated library DNA 1 (Fig. S1D) or selected aptamers (Fig. S3C) and H_2O_2 or BT as indicated in figures and incubated at 37°C for indicated times.

Following in vitro biotinylation, protein in lysates was digested with 4 µL proteinase K solution and 36 µL buffer ATL (Qiagen DNEasy Blood and Tissue DNA Extraction kit # 69054) by shaking at 56°C for 1 h with 1000 rpm shaking. 150 µL phenol:chloroform solution (VWR # 0883) was added and samples were subjected to vortex mixing. This was followed by 5 min centrifugation at 16,000 × g and the upper aqueous phase was collected. DNA was precipitated by the addition of 2.5 volume equivalents EtOH in the presence of 0.1 volume equivalent 3M NaOAc, and 1 µL glycogen solution (Roche # 10901393001) followed by chilling on dry ice for 15 min. DNA was pelleted by 15 min centrifugation at 16,000 × g. Pellets were washed once in 150 µL 70% EtOH and subjected to centrifugation again at 16,000 × g for 5 min. Supernatants were discarded and DNAs were resuspended in 15 µL water.

For gel shift assays, 5 µL Streptavidin solution (182 µM, Thermo # 434302) was then added to each 15 µL sample and mixed by pipetting before incubating at room temperature for 30 min. This 20 µL total volume was combined with loading dye (Invitrogen # 10482035). Samples with loading dye (8 µL) were then loaded into native 10% polyacrylamide gel (19:1) and subjected to electrophoresis at 24 V/cm for 30 min.

For qPCR detection assays, M-270 Streptavidin Dynabeads beads (Invitrogen # 65305) were used to capture biotinylated oligonucleotides. 50 µL of stock bead solution was transferred to a microcentrifuge tube and combined with 1 mL 1× Bind and Wash (B&W) buffer (10 mM Tris-HCl, pH 7.5, 1 mM EDTA, 2M NaCl, 0.1% Tween-20). Beads were placed on a magnetic stand for 1 min to separate from buffer and supernatant was discarded, followed by two washes in 200 µL 1× B&W buffer. Beads were finally resuspended in 50 µL 2× B&W buffer and combined with 50 µL isolated nucleic acids with thorough mixing by pipetting and incubated together at room temperature for 1 h with intermittent mixing as beads settled. Following bead capture, beads were again placed on a magnet stand and unbound DNAs were discarded. Beads were then extensively washed to remove any remaining unbound DNAs by the following steps: twice with 200 µL 1× B&W buffer (Wash A), once with 200 µL 0.1 M NaOH (Wash B), twice with Wash A, twice with Wash B, twice with Wash A, and once in water. Well-washed beads were resuspended in 50 µL water. M270 Dynabeads with bound oligonucleotides served as templates for qPCR analysis as done in the main methods section. Two biological replicates were performed for each DNA.

1.4 Thermal Denaturation Analysis

Thermal denaturation of folded oligonucleotide structures was analyzed in 140 mM Na^+, 4.5 mM K^+, 1.5 mM Ca^{2+}, 1 mM Mg^{2+} at 1 µM oligonucleotide concentration. 1-cm path length quartz cuvettes were loaded with 1 mL buffer before adding 10 µL of oligonucleotide solutions for analysis. Oligonucleotides were folded by heating to 90 ºC for 5 min and snap cooling on ice. Absorbance measurements were then obtained using a Cary 300 Bio UV-Vis spectrophotometer thermal program over the temperature range

of 4-90 ºC, at increments of 1 ºC, and a heating rate of 1 ºC/min. Melting point determination was performed using first derivative calculations in Microsoft Excel.

1.5 Aptamer Stability in Cell Lysate

Whole cell extract was prepared from approximately 10 million HEK293T cells. Two 10 cm plates of cells were grown to confluence, then washed twice with 10 mL fresh DMEM and scraped into Falcon tubes. Cells were subjected to centrifugation at 1000 × g and supernatant removed. Cells were resuspended in 200 µL ice-cold lysis buffer per 10 cm plate (20 mM Tris-Cl, pH 8.0; 100 mM KCl; 10% v/v glycerol; 1 mM DTT) and subjected to sonication 3 times for 10 s each, with resting on ice for 1 min between. Cell extract was then subjected to centrifugation at 15,000 × g for 20 min at 4ºC and supernatant was saved. Protein concentration was determined using a Qubit 3 Fluorometer (Invitrogen) according to manufacturer's instructions.

Oligonucleotides labeled with 5' FAM were prepared at 200 nM in PBS supplemented with 1× NEB CutSmart buffer (NEB # B7204S), 1 mM $CaCl_2$, and 1 mM $MgCl_2$, and were folded by heating to 90ºC and snap cooling on ice. Whole cell extract (50 µg) was added to 50 µL of folded oligonucleotide on ice. Samples were then transferred for incubation at 37°C for various lengths as indicated. Reactions were stopped by freezing samples on dry ice. LDS buffer (10 µL of 5X stock) was added to the frozen samples, and then samples were heated to 90°C for 5 min to inactivate enzymes. Samples were subjected to electrophoresis on a denaturing 10% polyacrylamide gel (19:1 acrylamide:bisacrylamide, 7.5 M urea) with 0.5× TBE running buffer at 26 V/cm for 30 min. Fluorescent oligonucleotides were imaged using an Amersham Typhoon fluorometric imager.

1.6 Aptamer Stability in Cell Culture Media

Oligonucleotide stocks were prepared at 2 µM concentration in PBS supplemented with 1 mM $MgCl_2$. Conditioned media was obtained from an overnight cell culture. Oligonucleotide stock (5 µL) was added to 45 µL of fresh or conditioned medium and incubated at 37℃ for up to 3 h as indicated. Reactions were terminated by freezing on dry ice. For analysis, 2.5 µL of each reaction was mixed with 5 µL deionized formamide

and 2.5 µL 4× LDS loading buffer and heated to 90℃ for 5 minutes. Samples were subjected to electrophoresis through a denaturing 10% polyacrylamide gel (19:1 acrylamide:bisacrylamide, 7.5 M urea) with 0.5× TBE running buffer at 26 V/cm for 30 min. Fluorescent oligonucleotides were imaged using an Amersham Typhoon fluorometric imager.

1.7 Chemical and Enzymatic Secondary Structure Determination

Polyacrylamide gel purified oligonucleotides labeled with 5’ FAM were prepared at 2 µM concentration in PBS containing 1 mM $MgCl_2$ (or 8 M urea for $KMnO_4$ studies). Samples were heated to 90°C for 5 min to denature and then placed on ice.

For dimethyl sulfate (DMS) probing, aptamer solution (50 µL) was combined with 1 µL DMS Solution (Sigma Aldrich # D186309) and incubated at room temperature for 5 min. The reaction was quenched by addition of 100 µL stop solution [1 M β-mercaptoethanol, 1 M Tris-Cl pH 7.5, 1.5 M NaOAc, 50 mM $Mg(OAc)_2$, 1 mM EDTA], and then combined with 1 µL yeast tRNA (10 mg/mL) as carrier. Samples were precipitated by adding 1 mL EtOH and chilling on dry ice for 15 min. Samples were subjected to centrifugation at 17,000 × g for 15 min and DNA pellets were washed with 500 µL of cold 70% ethanol. Pellets were air dried and resuspended in 60 µL of 10 mM sodium phosphate buffer (pH 7.0) containing 1 mM EDTA. Samples were heated to 90°C for 15 min and then chilled on ice. A solution of 1 M NaOH (6 µL) was then added, and samples were heated to 90 °C for 30 min. Samples were then cooled and mixed with 5 µL glycogen (5 mg/mL stock). Samples were precipitated from 500 µL EtOH as previously described and then subjected to centrifugation at 17,000 × g for 15 min and DNA pellets were washed with 250 µL cold 70% EtOH. Pellets were then air dried and resuspended in 20 µL water. For sample analysis by denaturing polyacrylamide gel electrophoresis, 1 µL of sample was combined with 7 µL deionized formamide and 1 µL loading dye before heating to 90°C for 5 minutes and cooling to room temperature.

For $KMnO_4$ reactivity studies, 40 µL aptamer solution was combined with 4 µL 160 mM $KMnO_4$ (Sigma Aldrich 223468) and incubated at room temperature for 1 min (native PBS) or for 10 s (denaturing urea). Reactions were quenched by addition of 5 µL

β-mercaptoethanol. Following quenching, samples were combined with 5 µL 500 mM EDTA, mixed, and placed on ice. Samples were then precipitated by adding 1 mL EtOH and processed as described above. Pellets were air dried and resuspended in 150 µL 10% (v/v) piperidine (Sigma # 104094). Samples were heated to 90 °C for 30 min and frozen on dry ice. Solvent was then removed by lyophilization, and samples resuspended in 50 µL water, 1 µL of 3 M NaOAc, and 1 µL of glycogen (5 mg/mL). Samples were precipitated from 400 µL EtOH and processed as described above. Pellets were then air dried and resuspended in 20 µL water. For sample loading into polyacrylamide gel, 2 µL of sample were taken and combined with 7 µL deionized formamide and 1 uL loading buffer before heating to 90°C for 5 min and cooling to RT.

Samples (10 µL) from DMS and $KMnO_4$ probing studies were subjected to electrophoresis through 16% denaturing polyacrylamide (19:1 acrylamide:bisacrylamide) sequencing gels containing 7.5 M urea in 0.5× TBE running buffer. Electrophoresis was for 2.5 h at 26 V/cm with drying and imaging on a Amersham Typhoon fluorimeter to detect oligonucleotide fluorescence.

For enzymatic probing, fluorescent oligonucleotides were prepared at 2 µM concentration in 1× Mung Bean nuclease reaction buffer (NEB # B0250). Samples were heated to 90ºC for 5 min and snap cooled on ice. Mung Bean nuclease (1 µL, NEB # M0250S) was added per 200 µL reaction. Samples were mixed by pipette and then incubated at 30ºC for 60 min. Samples were transitioned to ice, combined with 20 µL 3M NaOAc and 4 µL of salmon testes DNA (5 mg/mL), and mixed by vortex. Nucleic acid was precipitated by the addition of 1 mL of 100% EtOH and chilling on dry ice for 15 min. Samples were centrifuged at 17,000 × g for 15 min, supernatant removed, and pellets washed with 500 µL ice-cold 70% EtOH. Water (15 µL) and deionized formamide (45 µL) were then added to re-dissolve DNA and samples were heated to 90 °C for 5 min to denature. Samples (10 µL) from DMS and Mung Bean nuclease studies were subjected to electrophoresis through 16% denaturing polyacrylamide (19:1 acrylamide:bisacrylamide) sequencing gels (7.5 M urea) in 0.5× TBE running buffer. Electrophoresis was for 3.5 h at 26 V/cm with drying and imaging on an Amersham Typhoon fluorimeter to detect fluorescence of FAM-labelled oligonucleotides.

1.8 Aptamer-Protein Interaction Analysis

Eluted aptamer binding proteins were solubilized in lysis buffer (50mM TEAB pH 8.5, 5% SDS) and then subjected to in-solution trypsin digestion on a S-trap micro column (Protifi, Farmingdale, NY) following the manufacturers protocol. The resulting peptides were analyzed by LC-MS/MS on a Thermo Scientific Exploris 480 mass spectrometer coupled to a Thermo Ultimate 3000 RSLCnano HPLC system. The peptides from each sample were loaded onto a Halo C18 2.7μm EXP stem trap (Optimize Technologies, Oregon City, OR) and were separated by chromatography using 0.1 % formic acid in both the A solvent (98%water/2%acetonitrile) and B solvent (80% acetonitrile/10% isopropanol/10% water), with a 3%B to 40%B gradient over 120 minutes at 400 nl/min through a hand-packed PicoFrit 100um x 40cm column with Agilent Acclaim 2.2um C18 packing. The mass spectrometric data were acquired on the Exploris 480 mass spectrometer in a data dependent acquisition mode with a 2 second cycle time between the MS survey scan from 340-1800 m/z at resolution 120,000 (at 200m/z), followed by HCD MS/MS scans at resolution 15,000 with a NCE setting of 30 and the isolation width set to 1.2 m/z, in which ions with charge states of 2 to 4 were selected for MS/MS, and placed on an exclusion list for 30 seconds. The normalized AGC target settings were 300% for the MS and 80% for MS/MS scans with max ion inject times of 50 ms and 100 ms respectively. The mass spectrometric raw data was searched against the Swissprot human database (ver. 2022_01) using Andromeda search algorithm on the MaxQuant proteomics analysis platform (ver. 1.6.17.0) (PMID: 19029910). The search parameters were set as followed: a maximum of two missed cleavages, carbamidomethylation at cysteine as a fixed modification and oxidation at methionine and protein N-terminal acetylation as variable modifications. The first search and main search for MS/MS were set to 20 and 4.5 ppm, respectively. The maximum modifications per peptide was set to 5, and the maximum charge was set at 7. The revert type of the target–decoy analysis was chosen. The peptide spectrum match false discovery rate (FDR), protein FDR and the site decoy fraction were set to 0.01. The minimum peptide length was set to 7. The minimum unique and razor peptides for identification was set to 1. A label-free quantitation method iBAQ (intensity-based

absolute quantitation (PMID: 21593866)) was used to quantify the proteins identified across all the samples. iBAQ values were median normalized and a t-test performed for fold change comparisons.

Proteins identified by crosslinking with aptamer but not found by crosslinking to a negative control oligonucleotide were selected for Gene Ontology enrichment analysis. To ensure that genuine aptamer-specific protein interactions were considered, only proteins identified by at least two peptide counts in each of three biological replicates were used. Proteins with any peptides detected in negative control replicates were excluded to facilitate stringent analysis. Unique interaction cutoffs did not require unique peptide identification across replicates. These cutoffs resulted in the identification of 204 proteins unique to DNA 8, 100 proteins unique to DNA 10, and 142 proteins unique to DNA 14. “Shared” proteins were those present in multiple biological replicates for both aptamer and negative control DNA 15 with no statistically significant difference in fold change between samples (FDR-corrected $p > 0.05$) Gene Ontology enrichment analysis (PMID: 7779012) was performed using GO web portal using release 2022-07-01 (http://release.geneontology.org/2022-07-01/index.html).

2.0 Supplemental Data

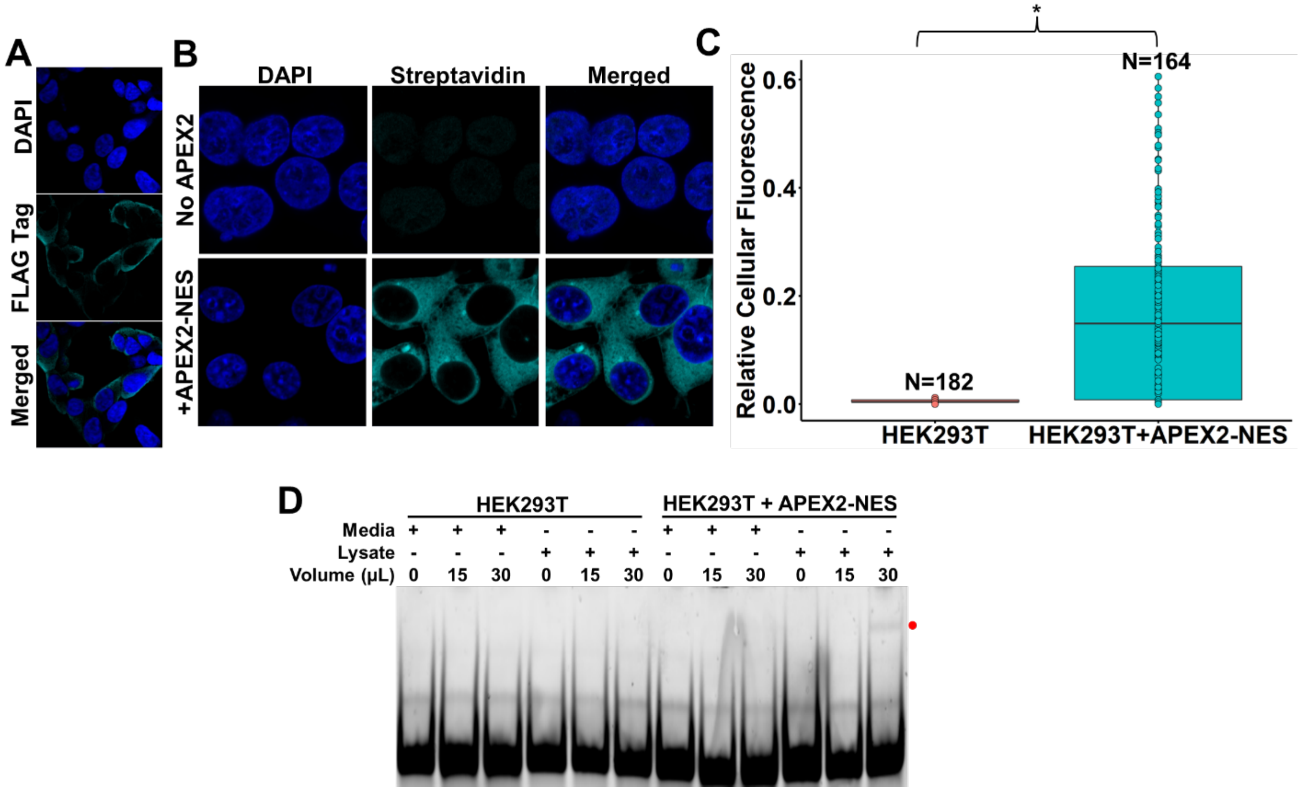

Figure S1. Confirmation of APEX2-NES localization and activity *in vivo* and *in vitro*. A) Stably-expressed FLAG-tagged APEX2-NES is specific to cytosol and excluded from regions of nuclear DAPI staining. Images collected at 100× magnification. B) HEK293T cells stably expressing APEX2-NES biotinylate cytosolic macromolecules in the presence of H_2O_2 and biotin tyramide (BT) as detected by staining with fluorescent streptavidin. HEK293T cells without engineered APEX2 do not induce detectable biotinylation. Images collected at 200× magnification. C) Quantification of per-cell fluorescence calculated from cells in panel B. Images collected at 20× magnification. D) Whole cell lysates of HEK293T cells stably expressing APEX2-NES biotinylate fluorescein-labeled oligonucleotides *in vitro* in the presence of H_2O_2 and BT. Gel shift (red dot) indicates binding of biotinylated oligonucleotide to streptavidin, present in all conditions together with BT and H_2O_2 as indicated. Background oligonucleotide biotinylation is not observed when APEX2-NES is absent from lysates. Media collected from overnight cell culture of both cell types is similarly inactive.

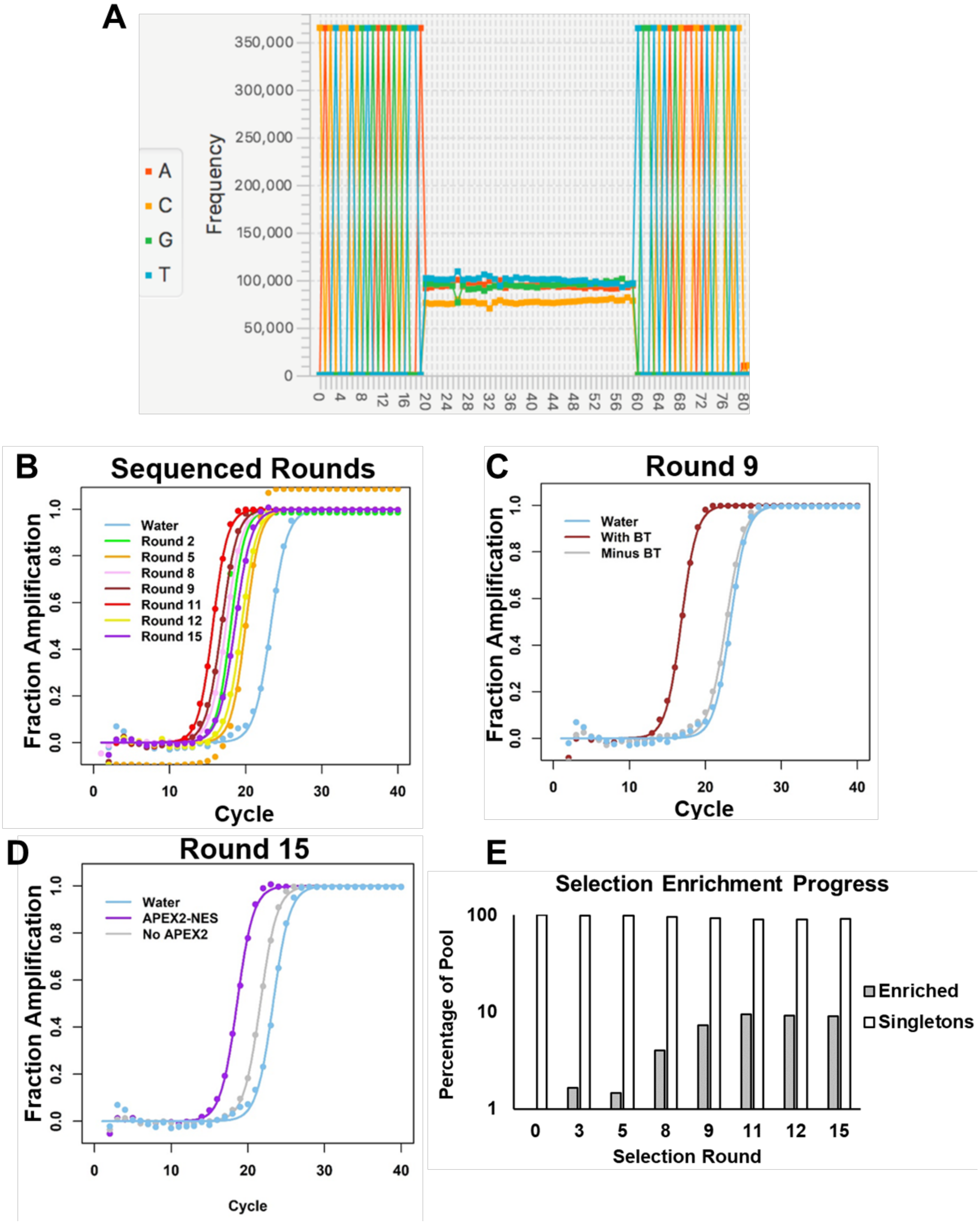
A
A
C
G
T
Frequency
350,000
300,000
250,000
200,000
150,000
100,000
50,000
0
0 4 8 12 16 20 24 28 32 36 40 44 48 52 56 60 64 68 72 76 80
B
Sequenced Rounds
Water
Round 2
Round 5
Round 8
Round 9
Round 11
Round 12
Round 15
Fraction Amplification
Cycle
C
Round 9
Water
With BT
Minus BT
Fraction Amplification
Cycle
D
Round 15
Water
APEX2-NES
No APEX2
Fraction Amplification
Cycle
E
Selection Enrichment Progress
Percentage of Pool
100
10
1
Enriched
Singletons
0 3 5 8 9 11 12 15
Selection Round

Figure S2. Selection progress monitored by qPCR. A) Assessment of base enrichment at each position of the naïve selection library reveals similar distribution of bases among every position in the 40 nt random region. The internal 40 nt random region is flanked by two constant 20 nt regions corresponding to forward and reverse primer homology regions. B) Normalized qPCR results demonstrating recovery above background signal ("water") at the indicated selection rounds. Changes in round-to-round recovery are partially attributable to variable amounts of aptamer library used. C) Parallel mock selections were performed at round 9 (amplified from round 8 recovered material) with and without biotin tyramide (BT) present in cell culture media. Library recovery was dependent on the addition of BT during selection. D) Parallel mock selections were performed at round 15 (amplified from round 14 recovered material) comparing cells with and without stable expression of APEX2-NES. E) Deep sequencing analysis reveals an increase in enriched aptamer clones over the course of the selection; approximately 10%of the aptamers sequenced in round 15 have at least one copy.

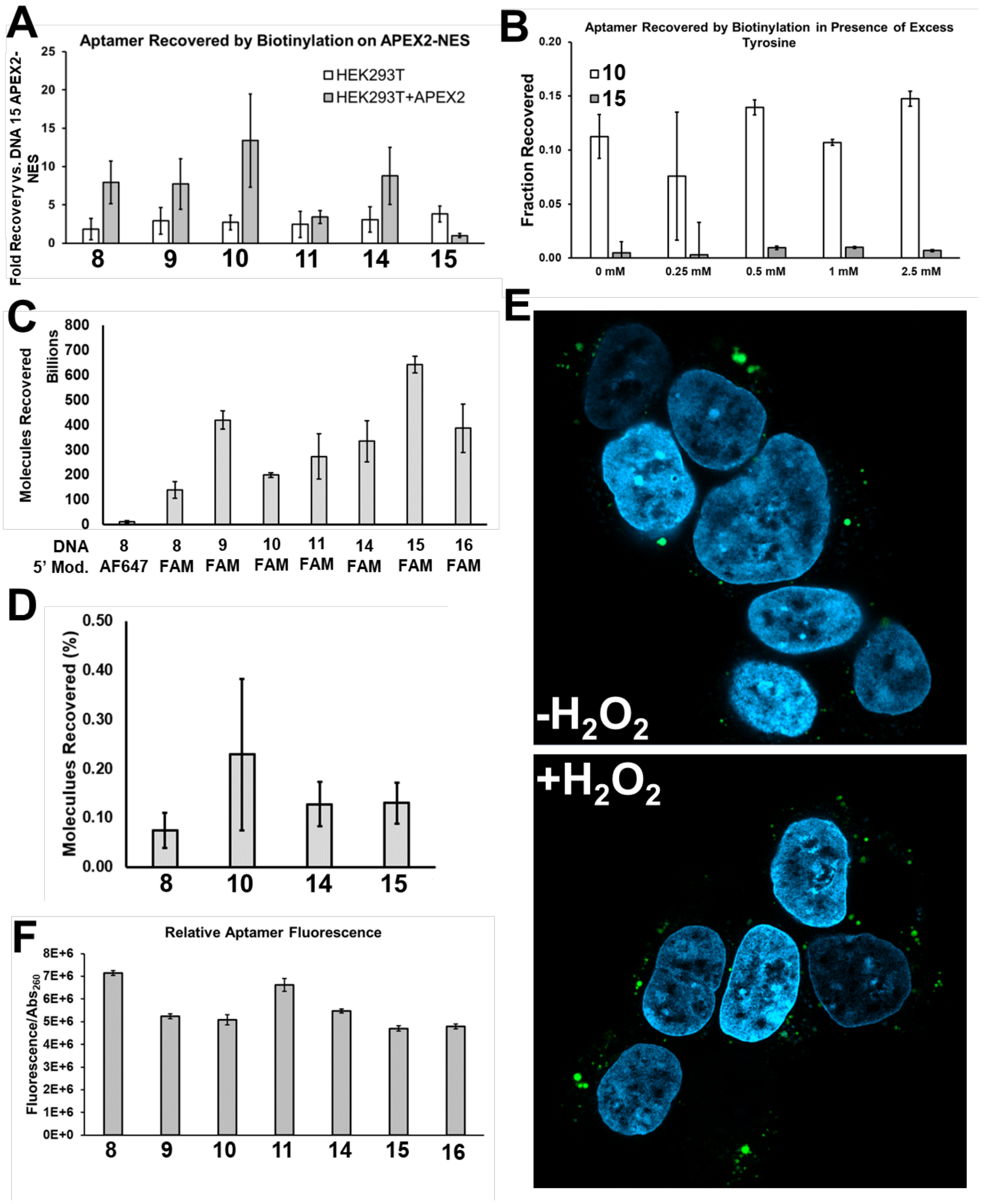

Figure S3. Control experiments for aptamer internalization assays and macropinosome stability in H_2O_2. A) An assay of cellular internalization and *in vivo* biotinylation by BT and H_2O_2 reveals that 4 of the

5 tested candidates are biotinylated within cells and can be captured with magnetic streptavidin beads. Capture is only possible when aptamers are incubated on cells expressing APEX2-NES. B) Tyrosine in cell culture at up to 12,500-fold molar excess relative to aptamer does not quench biotinylation or bead capture of DNA 10. Negative control DNA 15 is poorly recovered under all conditions. C) An assay of *in vitro* biotinylation, performed by capturing biotinylated DNA on streptavidin beads and quantifying recovery by qPCR, shows that DNA 8, 9, 10, 11, and 14 are not more efficiently biotinylated than negative controls DNA 15 and 16. DNA 8 cannot be biotinylated when a 5' FAM modification is replaced by a 5' AlexaFluor647. D) Selected 5' fluorescein-modified aptamers do not bind to M270 streptavidin Dynabeads under washing conditions used during selection, eliminating the possibility that enriched aptamers were selected for interactions with streptavidin. F) 5' AlexaFluor 647-modified DNAs 8-15 are labeled to similar degrees, demonstrated by relative fluorescence per A_{260nm} per sample. E) HEK293T cells are treated with to fluorescein-modified dextran (green) to label macropinosomes and challenged with H_2O_2 under SELEX conditions (100 mM for 1 min). This H_2O_2 exposure does not disrupt macropinosome stability, demonstrated by the presence of macropinosomes containing dextran. Images collected at 145× magnification. Cells were counterstained with DAPI (blue).

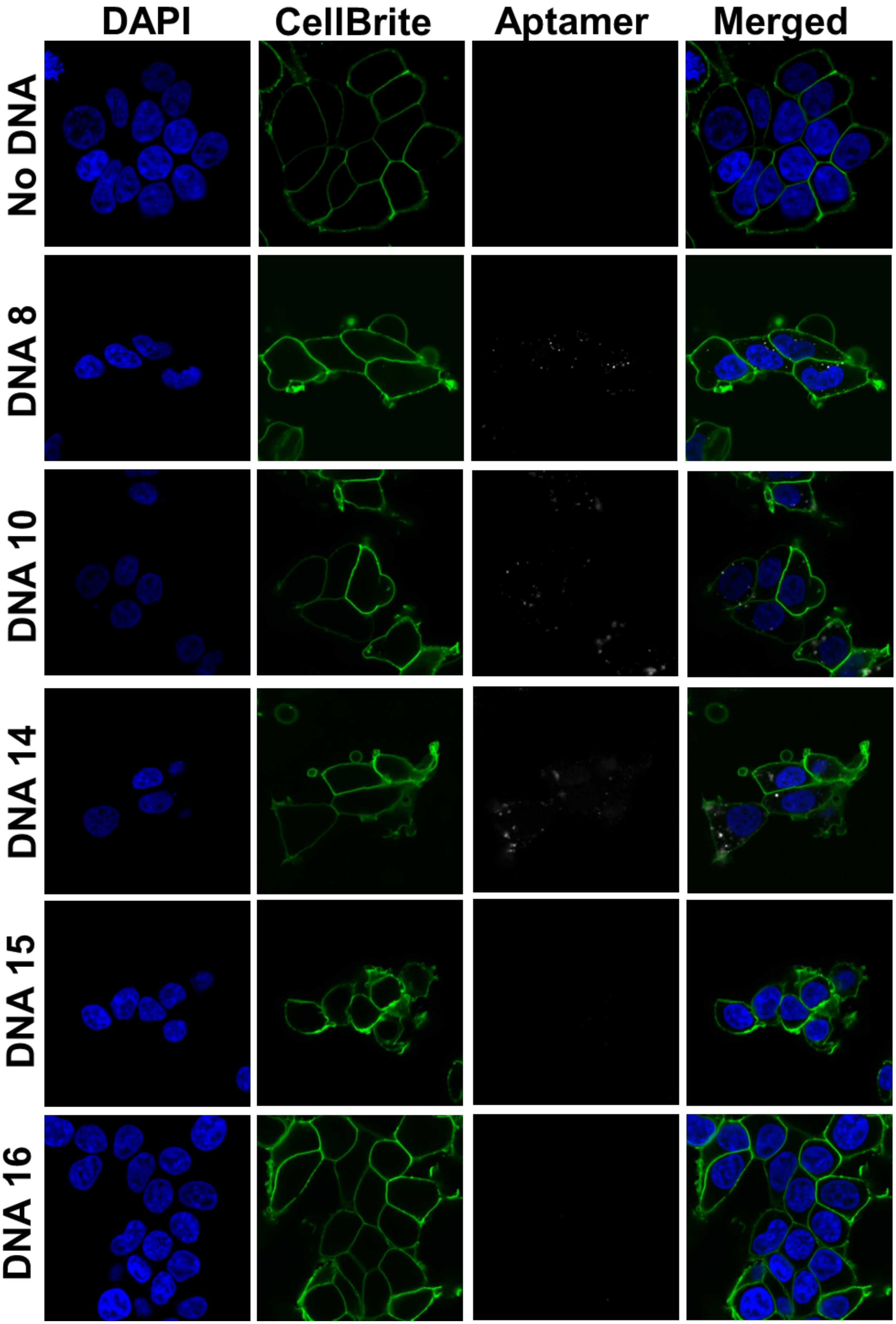
DAPI
CellBrite
Aptamer
Merged
No DNA
DNA 8
DNA 10
DNA 14
DNA 15
DNA 16

Figure S4. Confocal microscopy assessment of aptamer internalization in HEK293T cells. Fluorescently-labeled aptamers (5’ AlexaFluor 647, white signal) DNA 8, DNA 10, and DNA 14 are visible in fixed and stained cells whereas negative controls DNA 15 and DNA 16 are not. Distinct puncta are distributed throughout cells in all cases of positive uptake. Images collected at 100× magnification.

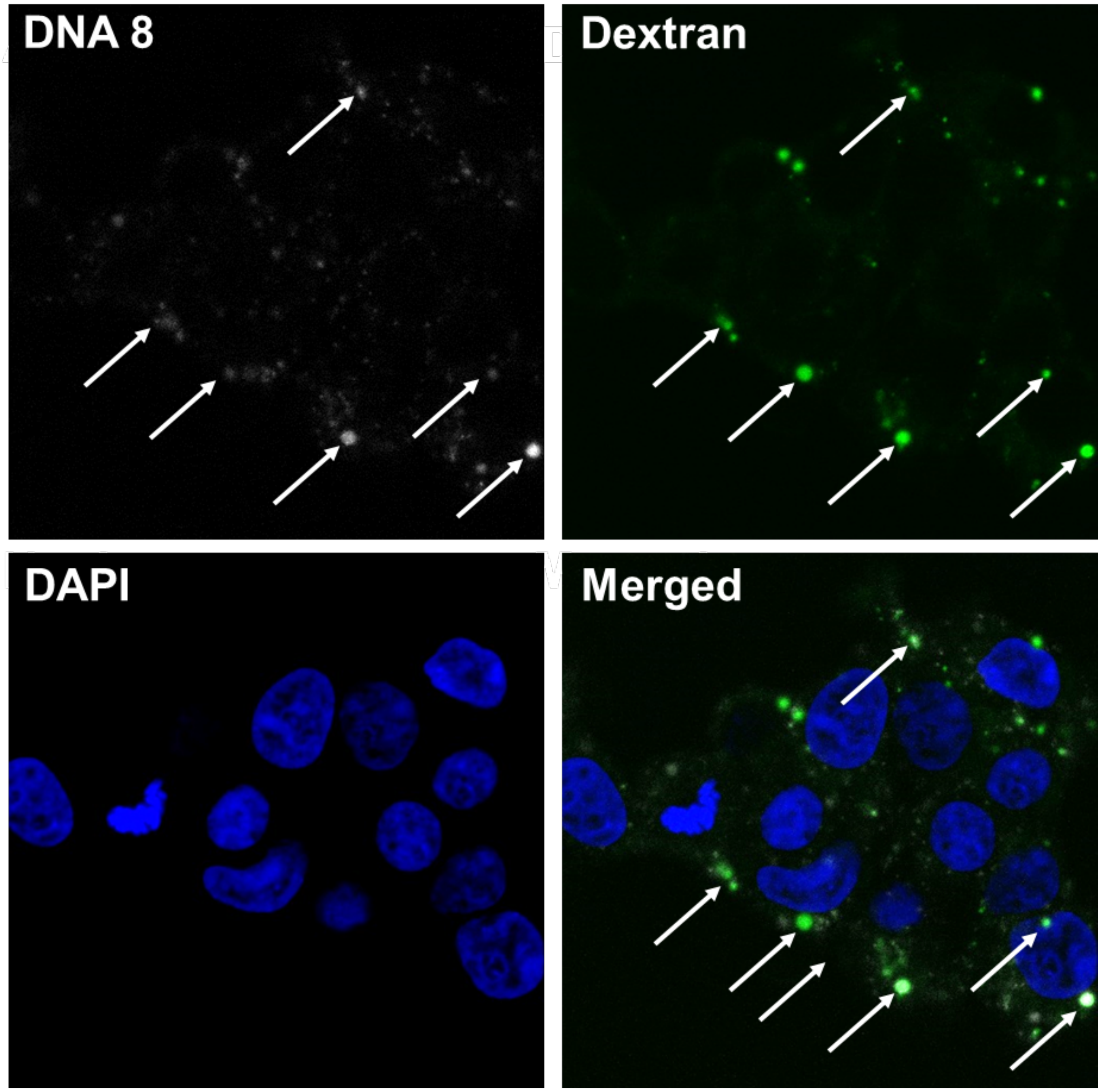

Figure S5. Confocal microscopy assessment of colocalization of DNA 8 and dextran. Fluorescently-labeled aptamer (5' AlexaFluor 647; white signal) DNA 8 incubated with HEK293T cells for 2 h in media with fluorescein-labeled 70 kDa dextran shows signal colocalization. Images collected at Images collected at 100× magnification.

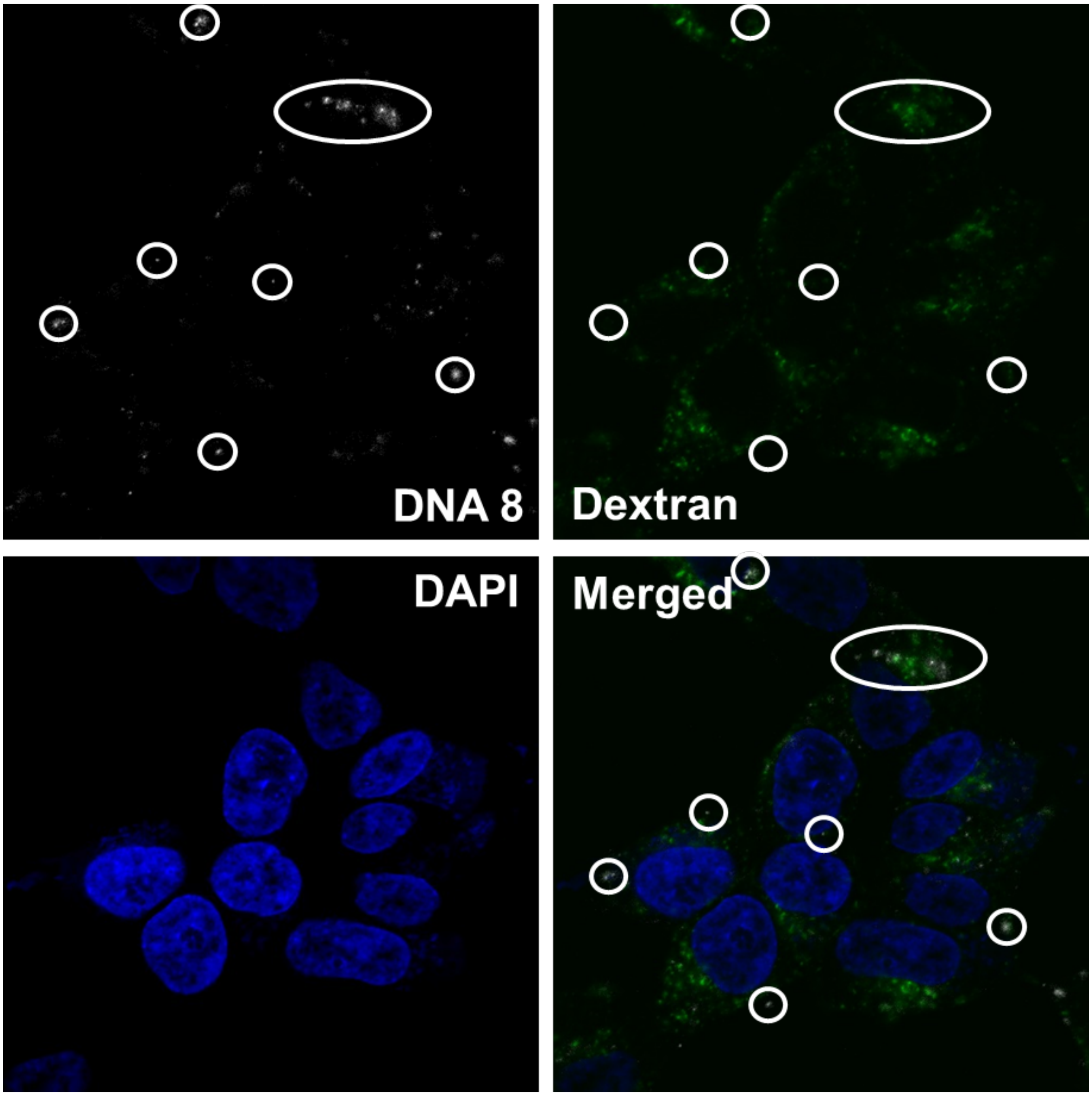

Figure S6. Confocal microscopy assessment of colocalization of DNA 8 and transferrin. Fluorescently labeled (5’ AlexaFluor 647, white signal) DNA 8 incubated on HEK293T cells for 2 h in media with fluorescein-labeled transferrin shows no signal colocalization (white circles). Images collected at 100× magnification.

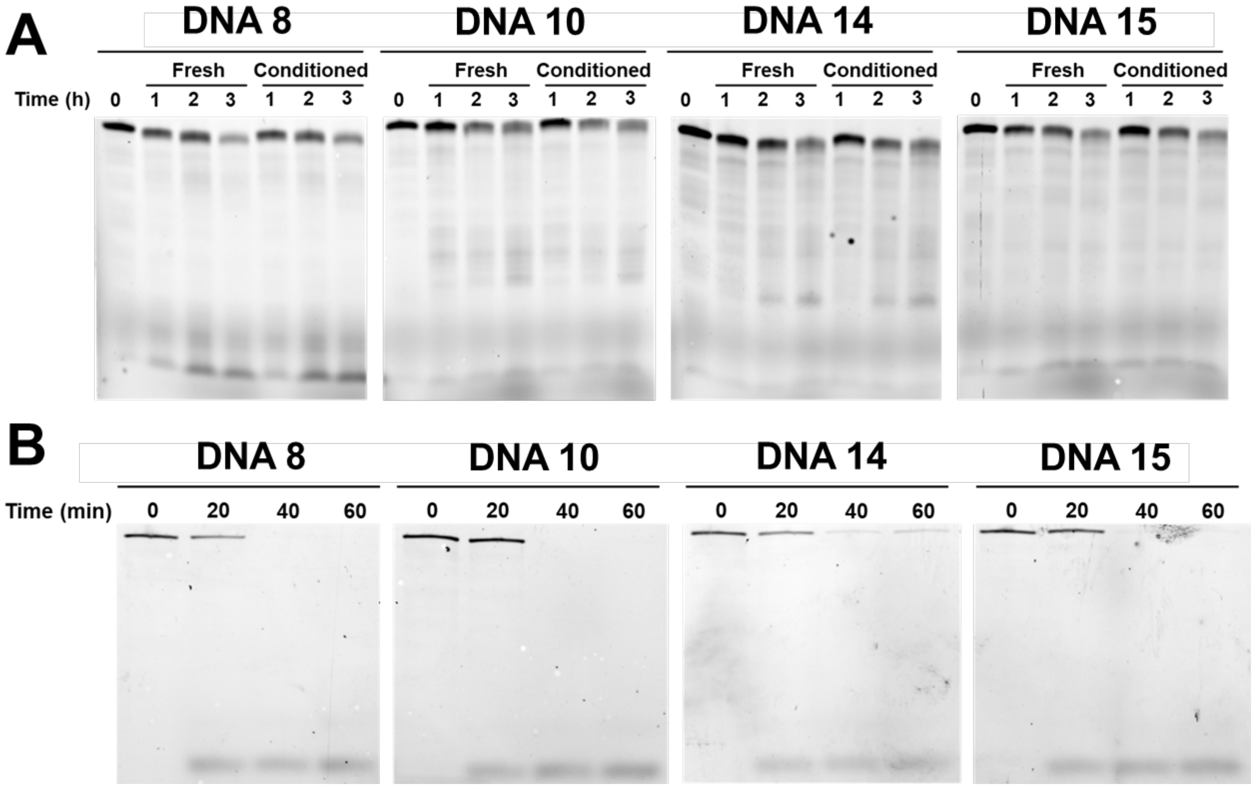

Figure S7. Nuclease stability assays. A) DNA 8, 10, and 14 along with negative control DNA 15 are similarly stable when challenged by nucleases from fresh cell culture media or conditioned media from cells grown overnight (up to 3 h at 37°C) B) DNA 8 and 10 and negative control DNA 15 are similarly stable when challenged by nucleases from whole cell lysates. DNA 14 is slightly more nuclease resistant (60 min). Incubation was at 37°C.

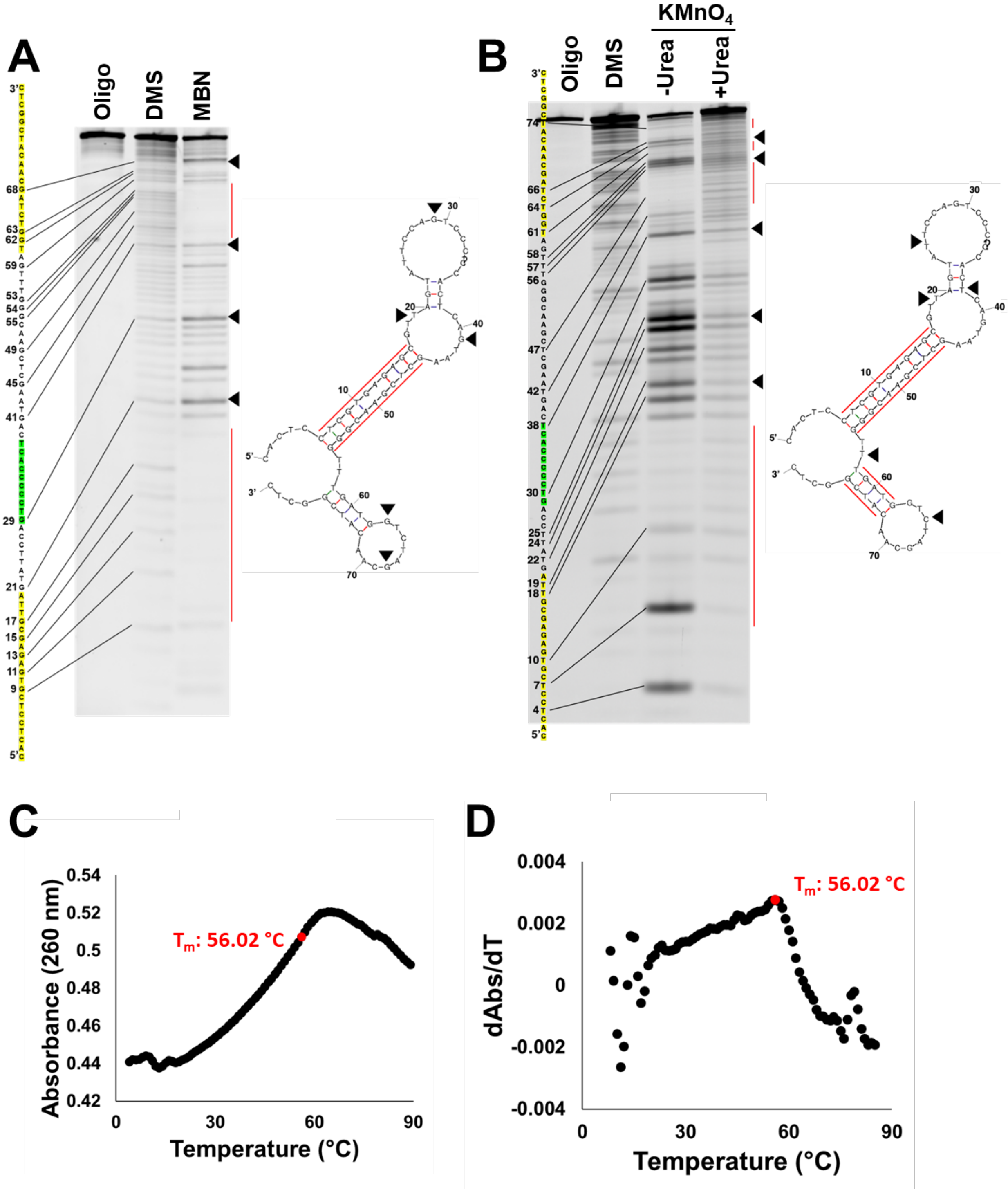

Figure S8. Structure probing and thermal melt denaturation of DNA 8. A) Denaturing gel electrophoresis of aptamer exposed to limiting Mung Bean nuclease (MBN) digestion, which preferentially occurs on single-stranded regions. B) Denaturing gel electrophoresis of aptamer cleaved by $KMnO_4$

reaction, which preferentially occurs at exposed thymine bases (compare native and denaturing conditions). A G>A dimethyl sulfate (DMS) sequencing ladder provide sequence markers. Black triangles indicate cleaved positions in agreement with the predicted secondary structure; red lines indicate protected positions in agreement with the predicted secondary structure. Yellow highlighting denotes constant flanking sequences; green highlighting denotes conserved 10 nt motif. C) Thermal melt denaturation from 0-90 °C identifies the melting temperature of DNA 8 as determined by the maximum of the first derivative (D).

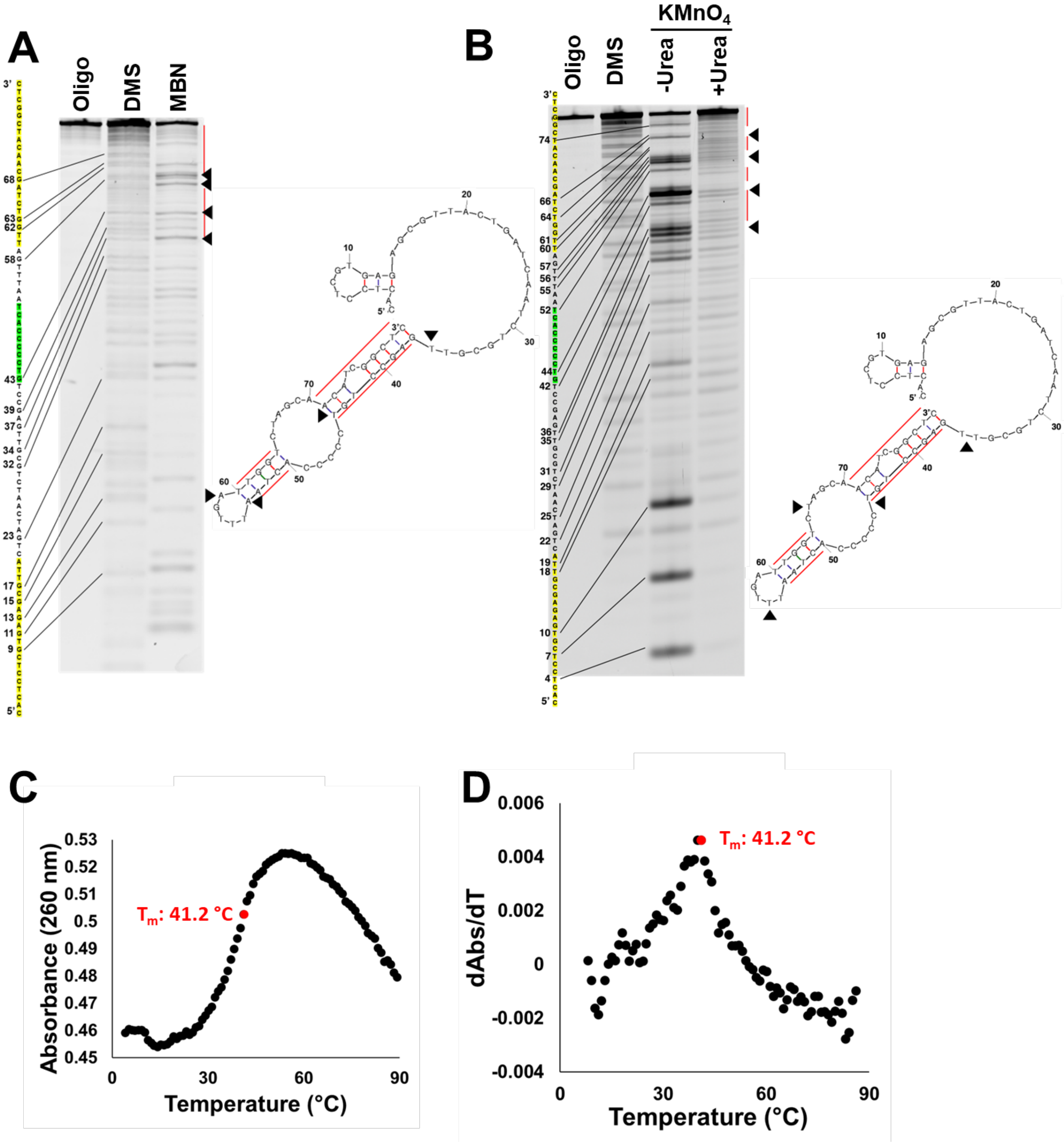

Figure S9. Structure probing and thermal melt denaturation of DNA 10. A) Denaturing gel electrophoresis of aptamer exposed to limiting Mung Bean nuclease (MBN) digestion, which preferentially occurs on single-stranded regions. B) Denaturing gel electrophoresis of aptamer cleaved by $KMnO_4$

reaction, which preferentially occurs at exposed thymine bases (compare native and denaturing conditions). A G>A dimethyl sulfate (DMS) sequencing ladder provide sequence markers. Black triangles indicate cleaved positions in agreement with the predicted secondary structure; red lines indicate protected positions in agreement with the predicted secondary structure. Yellow highlighting denotes constant flanking sequences; green highlighting denotes conserved 10 nt motif. C) Thermal melt denaturation from 0-90 °C identifies the melting temperature of DNA 10 as determined by the maximum of the first derivative (D).

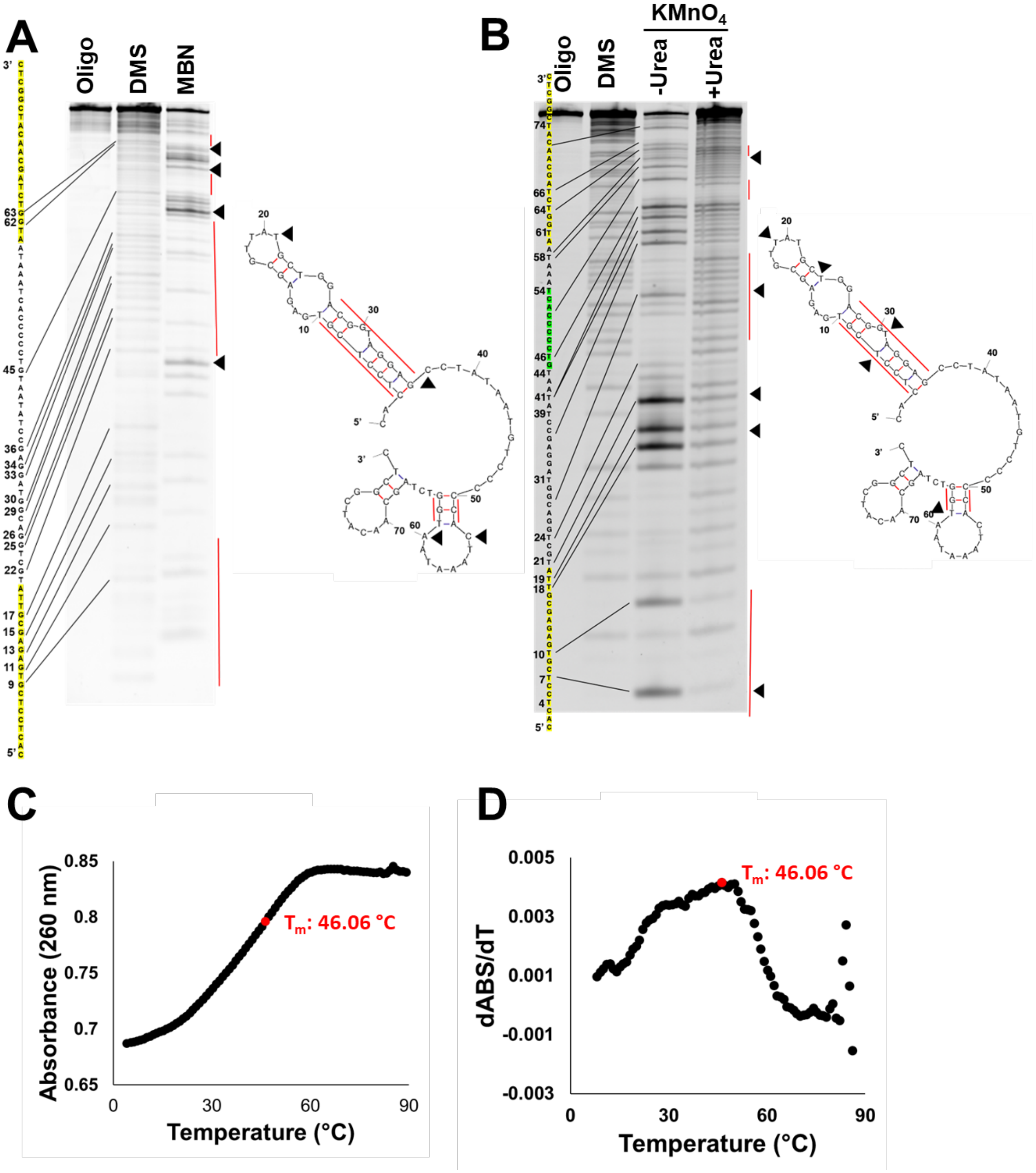

Figure S10. Structure probing and thermal melt denaturation of DNA 14. A) Denaturing gel electrophoresis of aptamer exposed to limiting Mung Bean nuclease (MBN) digestion, which preferentially occurs on single-stranded regions. B) Denaturing gel electrophoresis of aptamer cleaved by $KMnO_4$ reaction, which preferentially occurs at exposed thymine bases (compare native and denaturing

conditions). A G>A dimethyl sulfate (DMS) sequencing ladder provide sequence markers. Black triangles indicate cleaved positions in agreement with the predicted secondary structure; red lines indicate protected positions in agreement with the predicted secondary structure. Yellow highlighting denotes constant flanking sequences; green highlighting denotes conserved 10 nt motif. C) Thermal melt denaturation from 0-90 °C identifies the melting temperature of DNA 14 as determined by the maximum of the first derivative (D).

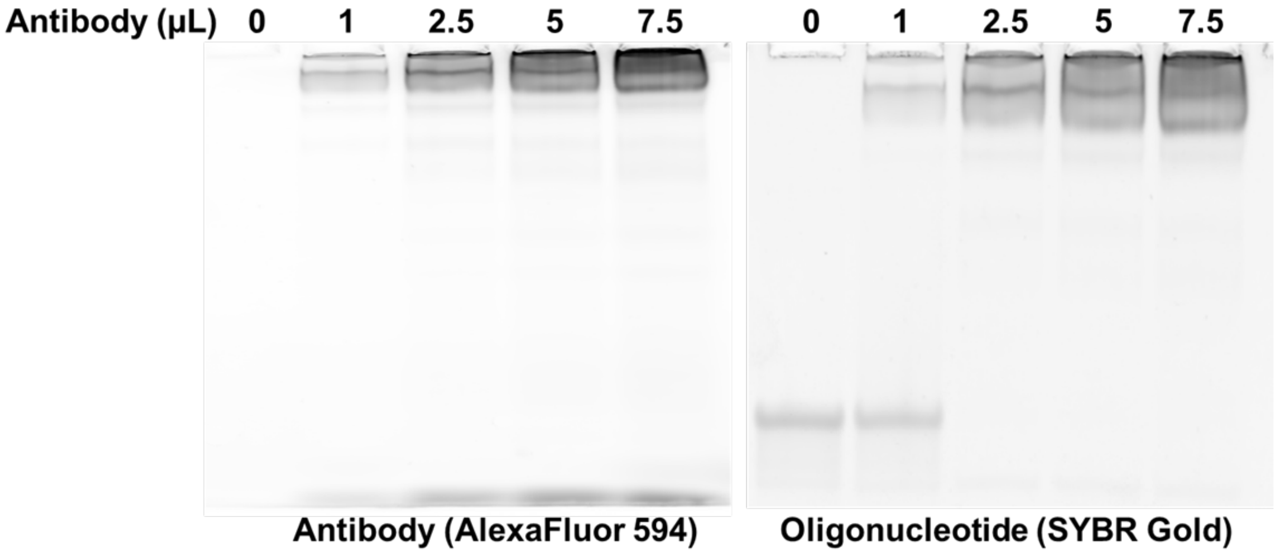

Figure S11. Anti-digoxigenin antibody conjugation to 5' digoxigenin-modified DNA 8. Native polyacrylamide gel electrophoresis of a fixed amount (10 pmol) of DNA 8 incubated with variable volumes of antibody demonstrating that oligonucleotides (detected by SYBR Gold) can be conjugated and gel shifted to co-localize with antibody-specific AlexaFluor 594 signal. 10 pmol of DNA 8 is entirely gel shifted when combined with 2.5 μL of antibody stock.

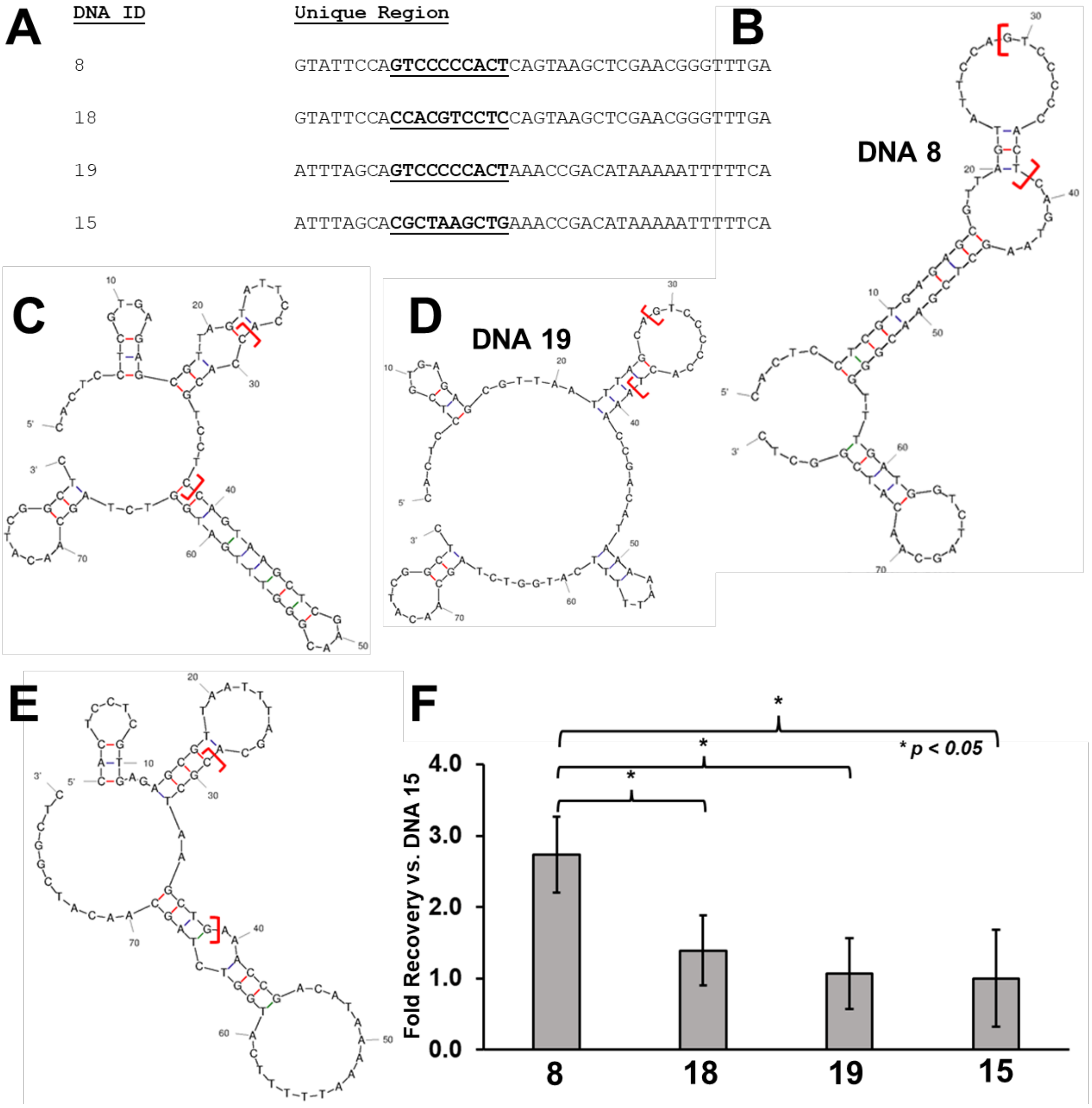

Figure S12. 10-nt motif GTCCCCCACT is necessary but not sufficient for internalization of DNA 8. A) Sequence comparison for unique regions of tested aptamers, with GTCCCCCACT motif or shuffled motif underlined. DNA 18 is generated by substituting the shuffled motif into DNA 8. DNA 19 was generated by substituting the original motif into negative control DNA 14. B-E) Predicted secondary structures of aptamers of interest. The motif GTCCCCCACT occurs in hairpin loops when present (B, D). F) Quantification of aptamer recovered by cell association assay relative to negative control DNA 14. Only DNA 8 is recovered above background.

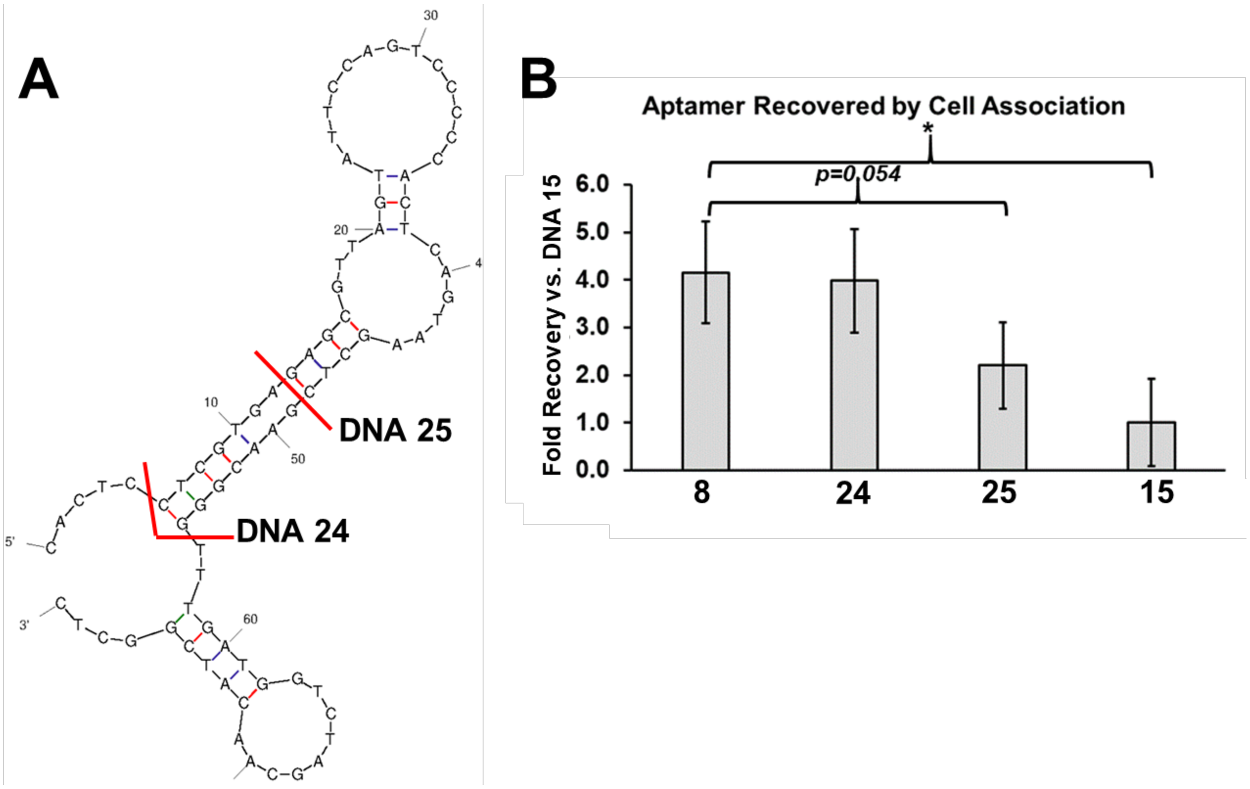

Figure S13. Truncating DNA 8 to remove portions of predicted secondary structure reveals core sequences required for activity. A) Predicted secondary structure of DNA 8 with red lines indicating where truncations were made to generate shortened molecules. B) Quantification of relative aptamer recovery versus negative control sequence DNA 14.

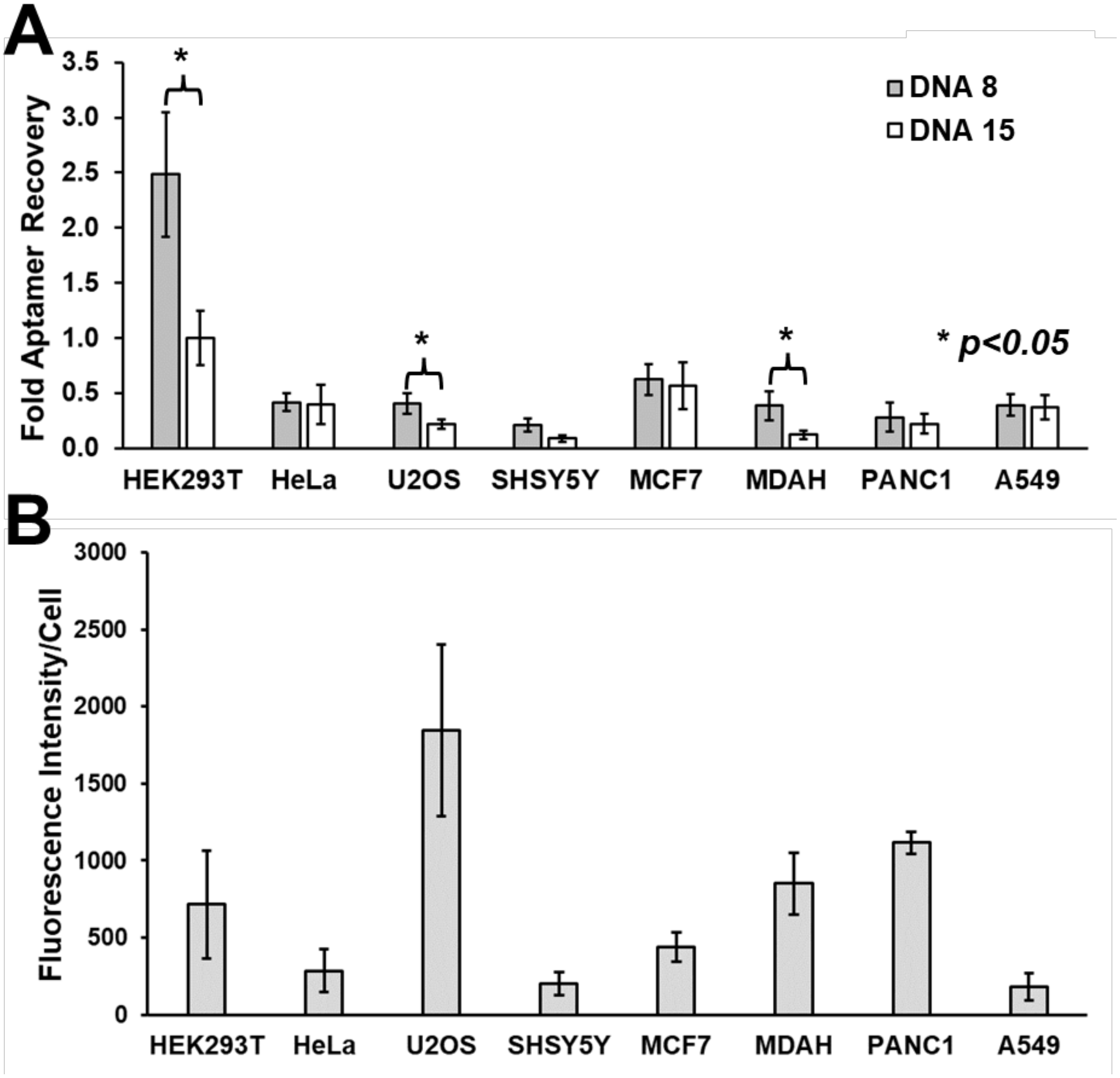

Figure S14. DNA 8 cell association is cell line-specific. A) DNA 8 association is higher than negative control DNA 14 only in HEK293T, U2OS, and MDAH cells. B) Relative macropinocytosis capacity of cell lines of interest measured. Macropinocytosis is measured by quantifying cellular internalization of fluorescently labeled 70 kDa dextran, a macropinocytosis-specific marker.

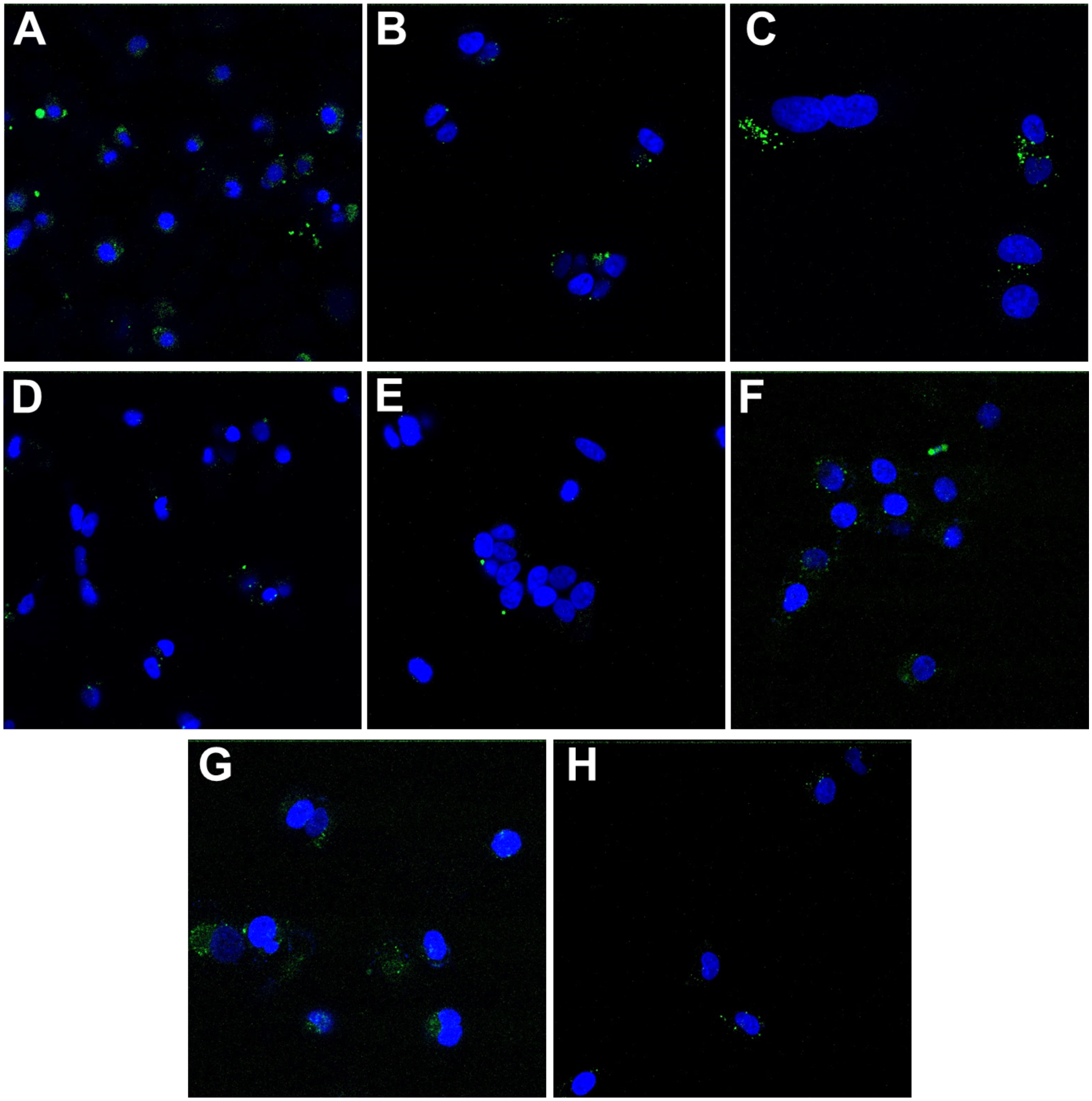

Figure S15. Visualization of 70 kDa dextran uptake in cell lines of interest. Fluorescein-labeled dextran (green) is used to measure relative macropinocytosis. Cell nuclei are identified by DAPI (blue) to enable quantification of the number of cells per field. Images are collected at 40× magnification. Cell lines of interest are A) HEK293T, B) HeLa, C) U2OS, D) SHSY5Y, E) MCF7, F) MDAH, G) PANC1, and H) A549.

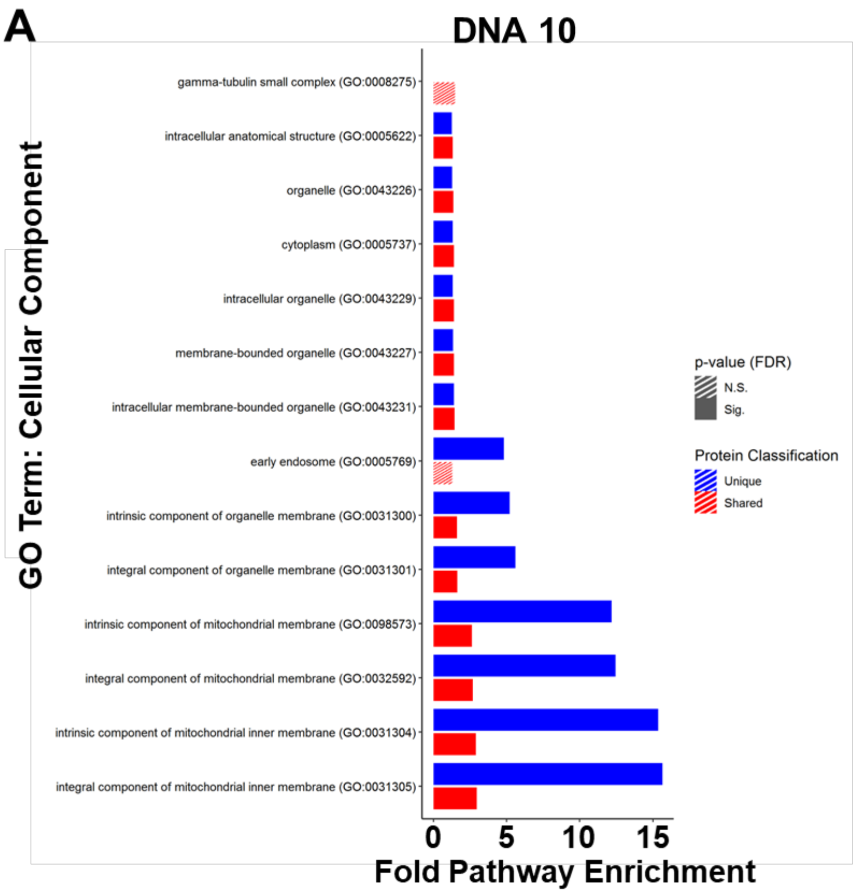
A
DNA 10
GO Term: Cellular Component
gamma-tubulin small complex (GO:0008275)
intracellular anatomical structure (GO:0005622)
organelle (GO:0043226)
cytoplasm (GO:0005737)
intracellular organelle (GO:0043229)
membrane-bounded organelle (GO:0043227)
intracellular membrane-bounded organelle (GO:0043231)
early endosome (GO:0005769)
intrinsic component of organelle membrane (GO:0031300)
integral component of organelle membrane (GO:0031301)
intrinsic component of mitochondrial membrane (GO:0098573)
integral component of mitochondrial membrane (GO:0032592)
intrinsic component of mitochondrial inner membrane (GO:0031304)
integral component of mitochondrial inner membrane (GO:0031305)
0
5
10
15
Fold Pathway Enrichment
p-value (FDR)
N.S.
Sig.
Protein Classification
Unique
Shared

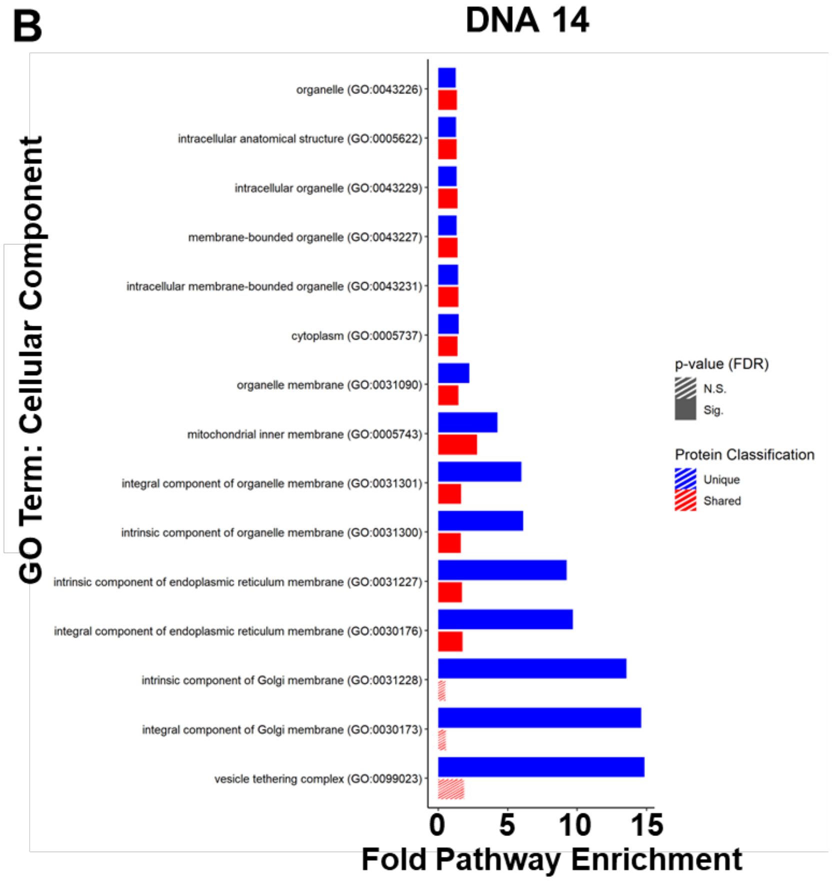
B
DNA 14
GO Term: Cellular Component
organelle (GO:0043226)
intracellular anatomical structure (GO:0005622)
intracellular organelle (GO:0043229)
membrane-bounded organelle (GO:0043227)
intracellular membrane-bounded organelle (GO:0043231)
cytoplasm (GO:0005737)
organelle membrane (GO:0031090)
mitochondrial inner membrane (GO:0005743)
integral component of organelle membrane (GO:0031301)
intrinsic component of organelle membrane (GO:0031300)
intrinsic component of endoplasmic reticulum membrane (GO:0031227)
integral component of endoplasmic reticulum membrane (GO:0030176)
intrinsic component of Golgi membrane (GO:0031228)
integral component of Golgi membrane (GO:0030173)
vesicle tethering complex (GO:0099023)
0
5
10
15
Fold Pathway Enrichment
p-value (FDR)
N.S.
Sig.
Protein Classification
Unique
Shared

Figure S16. Figure 5. Proteomic analysis of aptamer-cell interactions of DNA 10 and DNA 14. Gene Ontology (GO) overrepresentation analysis of proteins consistently interacting with A) DNA 10 or B) DNA 14, but not with negative control DNA 15, reveals highly enriched and statistically significant pathways among cellular component GO terms. For each enriched and significant GO term overrepresented among unique interactions (blue), proteins interacting with both aptamer and DNA 15 (red) are compared and statistical significance is assessed.

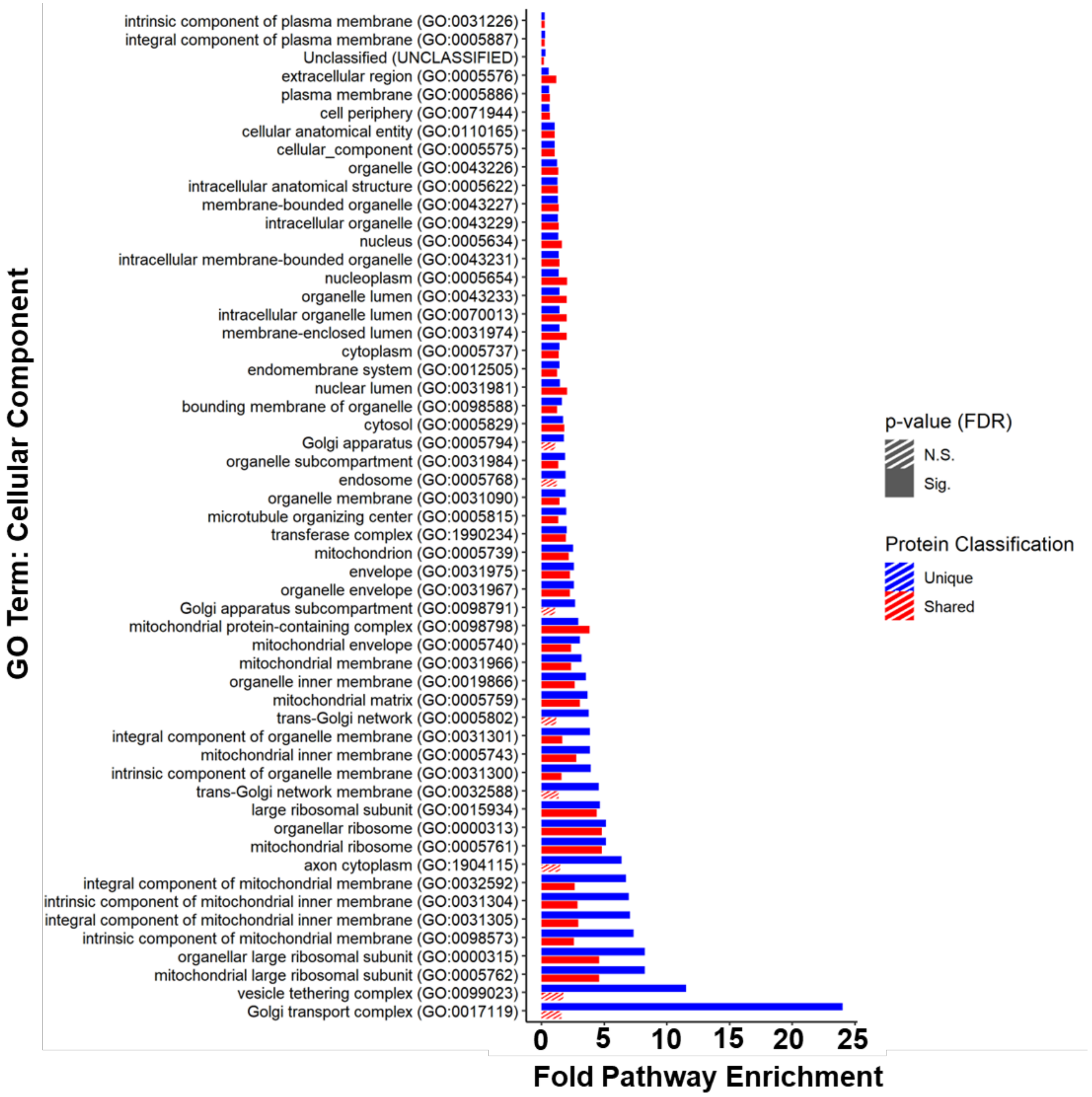

Figure S17. Proteomic analysis of aptamer-cell interactions of DNA 8. Gene Ontology (GO) overrepresentation analysis of proteins consistently interacting with DNA 8 but not with negative control DNA 15 reveals multiple highly enriched and statistically significant pathways among cellular component GO terms. For each enriched and significant GO term overrepresented among unique interactions (blue), proteins interacting with both DNA 8 and DNA 15 (red) are compared and statistical significance is assessed. Cellular component GO terms related to nonspecific extracellular aptamer-cell interactions are more enriched among shared interactions.

DNA ID	Internal Lab Serial Number	Sequence
1	LJM-6527	CACTCCTCGTGAGAGCGTTA(N_{40})TGGTCTAGCAACATCGGCTC
2	LJM-6528	/56-FAM/+CA+CT+CCTCGTGAGAGCGTTA
3	LJM-6529	A_{20}/iSp9//iSp9/GTACAGATCGTACTCAGACG
4	LJM-6530	CACTCCTCGTGAGAGCGTTA
5	LJM-6531	GTACAGATCGTACTCAGACG
6	LJM-6658	CACTCCTCGTGAGAGCGTTAATGTTGTAGGATCCGCATATCGTCCCCCACTCACATTAATTGGTCTAGCAACATCGGCTC
7	LJM-6659	CACTCCTCGTGAGAGCGTTACTGATCAATCTGCGTTGAACCTGTCCCCCACTAATTTGATTGGTCTAGCAACATCGGCTC
8	LJM-6660	CACTCCTCGTGAGAGCGTTAGTATTCCAGTCCCCCACTCAGTAAGCTCGAACGGGTTTGATGGTCTAGCAACATCGGCTC
9	LJM-6661	CACTCCTCGTGAGAGCGTTATGGTTGCTCGGCTTGCTCGTCTTATGTCCCCCACTACTCCTGGTCTAGCAACATCGGCTC
10	LJM-6662	CACTCCTCGTGAGAGCGTTACTGATCAATCTGCGTTGAGCCTGTCCCCCACTAATTTGATTGGTCTAGCAACATCGGCTC
11	LJM-6663	CACTCCTCGTGAGAGCGTTAGTCATGATACTAGAGCTTCATATATGTCCCCCACTCACTATGGTCTAGCAACATCGGCTC
12	LJM-6664	CACTCCTCGTGAGAGCGTTAGATGGTGCTACTTATCGTCCCCCACTGTTCTGGCCCGGCCTGGTCTAGCAACATCGGCTC
13	LJM-6665	CACTCCTCGTGAGAGCGTTAATGTTGCAGGATCCGCATATCGTCCCCCACTCACATTAATTGGTCTAGCAACATCGGCTC
14	LJM-6666	CACTCCTCGTGAGAGCGTTATGCTGGACGGTAGGAGCCTATAATGTCCCCCACTAAATAATGGTCTAGCAACATCGGCTC
15	LJM-6667	CACTCCTCGTGAGAGCGTTAATTTAGCACGCTAAGCTGAAACCGACATAAAAATTTTTCATGGTCTAGCAACATCGGCTC
16	LJM-6668	CACTCCTCGTGAGAGCGTTAAACCGATGCGTCTCGGTGAAGAAAGAAAGAGCCCTTTTGATGGTCTAGCAACATCGGCTC
17	LJM-6669	CACTCCTCGTGAGAGCGTTAGAGAGCACGATTAGCTAAGAGGACGAGTCCTCATCACGGGTGGTCTAGCAACATCGGCTC
18	LJM-6820	CACTCCTCGTGAGAGCGTTAGTATTCCACCACGTCCTCCAGTAAGCTCGAACGGGTTTGATGGTCTAGCAACATCGGCTC
19	LJM-6821	CACTCCTCGTGAGAGCGTTAATTTAGCAGTCCCCCACTAAACCGACATAAAAATTTTTCATGGTCTAGCAACATCGGCTC
20	LJM-6798	/5Dig/CACTCCTCGTGAGAGCGTTAGTATTCCAGTCCCCCACTCAGTAAGCTCGAACGGGTTTGATGGTCTAGCAACATCGGCTC
21	LJM-6799	/5Dig/CACTCCTCGTGAGAGCGTTACTGATCAATCTGCGTTGAGCCTGTCCCCCACTAATTTGATTGGTCTAGCAACATCGGCTC
22	LJM-6800	/5Dig/CACTCCTCGTGAGAGCGTTATGCTGGACGGTAGGAGCCTATAATGTCCCCCACTAAATAATGGTCTAGCAACATCGGCTC
23	LJM-6801	/5Dig/CACTCCTCGTGAGAGCGTTAATTTAGCACGCTAAGCTGAAACCGACATAAAAATTTTTCATGGTCTAGCAACATCGGCTC
24	LJM-6851	CCTCGTGAGAGCGTTAGTATTCCAGTCCCCCACTCAGTAAGCTCGAACGGG
25	LJM-6854	GAGCGTTAGTATTCCAGTCCCCCACTCAGTAAGCTC

Modification Code	Description
+	Locked nucleic acid
/56-FAM/	5' fluorescein
/iSp9/	Internal C9 spacer
/5Dig/	5' digoxigenin

Table S1. Identity number and sequences of oligonucleotides used in this study. All modifications are provided by Integrated DNA Technologies.

Aptamer Id	Sequence	% of Pool
993168	CACTCCTCGTGAGAGCGTTA**ATGTTGTAGGATCCGCATATCGTCCCCCACTCACATTAAT**TGGTCTAGCAACATCGGCTC	0.393% *
162699	CACTCCTCGTGAGAGCGTTA**CTGATCAATCTGCGTTGAACCTGTCCCCCACTAATTTGAT**TGGTCTAGCAACATCGGCTC	0.232% *
2710398	CACTCCTCGTGAGAGCGTTA**GTATTCCAGTCCCCCACTCAGTAAGCTCGAACGGGTTTGA**TGGTCTAGCAACATCGGCTC	0.065%
2283597	CACTCCTCGTGAGAGCGTTA**TGGTTGCTCGGCTTGCTCGTCTTATGTCCCCCACTACTCC**TGGTCTAGCAACATCGGCTC	0.058% *
2502867	CACTCCTCGTGAGAGCGTTA**CTGATCAATCTGCGTTGAGCCTGTCCCCCACTAATTTGAT**TGGTCTAGCAACATCGGCTC	0.047% *
2275328	CACTCCTCGTGAGAGCGTTA**GTCATGATACTAGAGCTTCATATATGTCCCCCACTCACTA**TGGTCTAGCAACATCGGCTC	0.029% *
2518842	CACTCCTCGTGAGAGCGTTA**GATGGTGCTACTTATCGTCCCCCACTGTTCTGGCCCGGCC**TGGTCTAGCAACATCGGCTC	0.028% *
4102400	CACTCCTCGTGAGAGCGTTA**ATGTTGCAGGATCCGCATATCGTCCCCCACTCACATTAAT**TGGTCTAGCAACATCGGCTC	0.024% *
2940286	CACTCCTCGTGAGAGCGTTA**ACGACTGTGCCCTAGAATTCCTGCTTGTCCCCCACTACTA**TGGTCTAGCAACATCGGCTC	0.018%
2618751	CACTCCTCGTGAGAGCGTTA**TGCTGGACGGTAGGAGCCTATAATGTCCCCCACTAAATAA**TGGTCTAGCAACATCGGCTC	0.017% *
5881794	CACTCCTCGTGAGAGCGTTA**ATGTTGTAGGACCCGCATATCGTCCCCCACTCACATTAAT**TGGTCTAGCAACATCGGCTC	0.014%
2312621	CACTCCTCGTGAGAGCGTTA**CAATAATGACTGTCCCCCACTCCCGTACTATTCACGTTAT**TGGTCTAGCAACATCGGCTC	0.012%
2455812	CACTCCTCGTGAGAGCGTTA**AGTCCCCCACTACACCCCTAGAGCACGTGAGAGAGTATTA**TGGTCTAGCAACATCGGCTC	0.012%
4461857	CACTCCTCGTGAGAGCGTTA**CTGATCAATCCGCGTTGAACCTGTCCCCCACTAATTTGAT**TGGTCTAGCAACATCGGCTC	0.012%
2563891	CACTCCTCGTGAGAGCGTTA**CTGATCAATCTGCGTCGAACCTGTCCCCCACTAATTTGAT**TGGTCTAGCAACATCGGCTC	0.010%
2977335	CACTCCTCGTGAGAGCGTTA**CAGTCGCGAGCTTGTCCCCCACTAGATCCTGGAGTGTAAA**TGGTCTAGCAACATCGGCTC	0.010%
3387382	CACTCCTCGTGAGAGCGTTA**CTGATCAATCTGCGTTGAACCCGTCCCCCACTAATTTGAT**TGGTCTAGCAACATCGGCTC	0.009%
5456569	CACTCCTCGTGAGAGCGTTA**ATGTTGTAGGATCCGCATATCGTCCCCCACTCACACTAAT**TGGTCTAGCAACATCGGCTC	0.009%
3205314	CACTCCTCGTGAGAGCGTTA**CTGACCAATCTGCGTTGAACCTGTCCCCCACTAATTTGAT**TGGTCTAGCAACATCGGCTC	0.008%
3011665	CACTCCTCGTGAGAGCGTTA**ATGTTGTAGGATCCGCATACCGTCCCCCACTCACATTAAT**TGGTCTAGCAACATCGGCTC	0.008%
2530876	CACTCCTCGTGAGAGCGTTA**GTACACGAAGGATACAGTCCCCCACTCCCCAATCTATACC**TGGTCTAGCAACATCGGCTC	0.007%
5475853	CACTCCTCGTGAGAGCGTTA**GTATCCCAGTCCCCCACTCAGTAAGCTCGAACGGGTTTGA**TGGTCTAGCAACATCGGCTC	0.007%
5945449	CACTCCTCGTGAGAGCGTTA**ATGTTGTAGGATCCACATATCGTCCCCCACTCACATTAAT**TGGTCTAGCAACATCGGCTC	0.007%
3284185	CACTCCTCGTGAGAGCGTTA**TAGCAATCTTGGTGCTAGCCTCGTCCCCCACTCGTCATA**TGGTCTAGCAACATCGGCTC	0.006%
5251809	CACTCCTCGTGAGAGCGTTA**ATGTTGTAGGATCCGCATATCGTCCCCCACTCACATTAGT**TGGTCTAGCAACATCGGCTC	0.006%
2194139	CACTCCTCGTGAGAGCGTTA**GAACACTCGAAAACACTATCTCGTCCCCCACTATTATGCG**TGGTCTAGCAACATCGGCTC	0.005%
3193442	CACTCCTCGTGAGAGCGTTA**TACGACCTATTCTTGAAACCGCACAGTCCCCTTTCACGTT**TGGTCTAGCAACATCGGCTC	0.005%
3838426	CACTCCTCGTGAGAGCGTTA**ATGTTGTAGGATCCGCATATCGTCCCCCACTCACATTGAT**TGGTCTAGCAACATCGGCTC	0.005%
5452941	CACTCCTCGTGAGAGCGTTA**TGGTTGCTCGGCCTGCTCGTCTTATGTCCCCCACTACTCC**TGGTCTAGCAACATCGGCTC	0.005%
5146460	CACTCCTCGTGAGAGCGTTA**ATGTTGTAGGATCCGCACATCGTCCCCCACTCACATTAAT**TGGTCTAGCAACATCGGCTC	0.005%
5957716	CACTCCTCGTGAGAGCGTTA**CTGATCAATCTGCGCTGAACCTGTCCCCCACTAATTTGAT**TGGTCTAGCAACATCGGCTC	0.005%
4802836	CACTCCTCGTGAGAGCGTTA**ATGTTGTAGGATCCGCATATCGTCCCCCACTCACATTAAA**TGGTCTAGCAACATCGGCTC	0.005%
4587916	CACTCCTCGTGAGAGCGTTA**GATGGTGCTGCTTATCGTCCCCCACTGTTCTGGCCCGGCC**TGGTCTAGCAACATCGGCTC	0.005%
3540716	CACTCCTCGTGAGAGCGTTA**ATGTTGTAGGATCCGCATATCGTCCCCCACTCACATAAAT**TGGTCTAGCAACATCGGCTC	0.004%
4979334	CACTCCTCGTGAGAGCGTTA**ATGTTGTAGGATCCGCATATCGTCCCCCACTTACATTAAT**TGGTCTAGCAACATCGGCTC	0.004%
3293859	CACTCCTCGTGAGAGCGTTA**ATGTTGTAGGATCCGCATATCGTCCCCCACTCGCATTAAT**TGGTCTAGCAACATCGGCTC	0.004%
5582185	CACTCCTCGTGAGAGCGTTA**ATGTTGTAGGATCCGCATATCGTCCCCCACTCACATCAAT**TGGTCTAGCAACATCGGCTC	0.004%
4654876	CACTCCTCGTGAGAGCGTTA**ATGTTGTAGGATCCGCGTATCGTCCCCCACTCACATTAAT**TGGTCTAGCAACATCGGCTC	0.003%
5951338	CACTCCTCGTGAGAGCGTTA**ATGTTGTAGGATCTGCATATCGTCCCCCACTCACATTAAT**TGGTCTAGCAACATCGGCTC	0.003%

*** Candidate aptamer***

Table S2. Forty most-enriched sequences by percentage of round 15 selection pool. The top 40 sequences detected in the final selection round are listed with random regions in bold text and the 10-nt motif GTCCCCCACT highlighted where present (yellow). 39 of 40 sequences include the motif, with one containing a truncated version (blue). Selected candidate aptamers are noted by a red asterisk.
